# Synthesis of New Spiro-Cyclopropanes Prepared by Non-Stabilized Diazoalkane Exhibiting an Extremely High Insecticidal Activity

**DOI:** 10.3390/molecules27082470

**Published:** 2022-04-12

**Authors:** Naoufel Ben Hamadi, Ahlem Guesmi

**Affiliations:** 1 Chemistry Department, College of Science, IMSIU (Imam Mohammad Ibn Saud Islamic University), P.O. Box 5701, Riyadh 11432, Saudi Arabia; amalkasme@imamu.edu.sa; 2 Laboratory of Heterocyclic Chemistry, Natural Products and Reactivity (LR11ES39), Faculty of Science of Monastir, UM (University of Monastir), Avenue of Environment, Monastir 5019, Tunisia; 3 Textile Engineering Laboratory, Higher Institute of Technological Studies of Ksar Hellal, UM (University of Monastir), Monastir 5000, Tunisia

**Keywords:** 1,3-dipolar cycloaddition, photolysis, 1*H*-pyrazole, spiro-cyclopropane, docking, insecticides

## Abstract

The synthesis of new insecticidal *gem*-dimethyspiro-cyclopropanes derived from pyrrolidine-2,3-dione have been described, and their biological effect against different insect species has been evaluated. The presented results demonstrate the excellent insecticidal activity of cyclopropane **5c** against *Aedes aegypti* and *Musca domestica*. Cyclopropane **5c** showed the quickest knockdown and the best killing against *Aedes aegypti* and *Musca domestica* compared to *trans*-chrysanthemic acid and pyrethrin. The biological results of the high insecticidal activity were confirmed by the results of docking. This is evident in the binding affinity obtained for cyclopropane **5c**, indicating good binding with an important active amino acid residue of the 5FT3 protein.

## 1. Introduction

**Figure 1:**
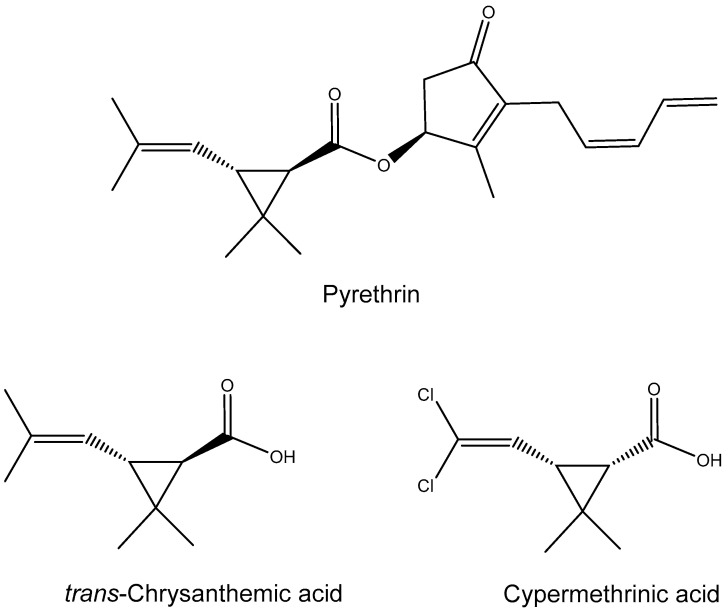
Structures of the most active cyclopropane derivatives.

Cyclopropanes are one of the best-known strained rings, having attracted the attention of major researchers for over a century due to their powerful and unique reactivity. They not only exist in many natural products [1,2,3,4], but have also been frequently used in many disciplines such as organic synthesis, materials science and biology and as building blocks for general-purpose materials. During the last decade, much research has been carried out to advance original regio-chemo and stereoselective approaches for the preparation and conversions of cyclopropane derivatives [5,6,7,8,9,10]. This research has generated considerable interest since fragments of cyclopropane are present in the structures of numerous biologically active substances, such as anticancer, antibiotics and antimycotic arrangements, regulators of plant development and maturation of fruits and insecticides [11,12]. Chrysanthemic acid and pyrethrins, insecticides isolated from the flower Chrysanthemum cinerariaefolium, are traditionally cited as examples of natural bioactive cyclopropanes. Their 2-vinylcyclopropanecarboxylic structural unit has been a good source of inspiration for the development of other synthetic cyclopropane insecticides for phytopharmaceutical use, such as deltamethrin (Roussel-Uclaf) (Figure 1) [13,14,15].

The application of spiro-cyclopropane structures in drug discovery has seen a dramatic increase in attention in recent years, alongside major developments in their synthetic chemistry [16]. Various biological activities have also been described for spiro-cyclopropane derivatives, such as anti-inflammatory and cytotoxic activity [17]. The consideration of compounds that include this structure has not been limited to molecules of synthetic origin but rather has been focused on natural products because this functionality has been described in numerous naturally occurring compounds. In the case of spiro-cyclopropanes destined for heterocycles, there are few reports of biological activity, especially the cyclopropane derivatives, which have been used as fungicides, pesticides and insecticides [18,19].

Photochemical diazotization of 1-pyrazoline derivatives is a widely used process in organic preparation to synthesize cyclopropane derivatives [20,21]. Pyrazoline derivatives are presently formed by 1,3-dipolar cycloaddition reactions of diazoalkanes to electron-deficient alkenes [22,23,24,25]. The current work describes the synthesis of spiro-cyclopropane derivatives via the photolysis of pyrazolines obtained by the cycloaddition of diazopropane with (*E*)-4-arylidene-pyrrolidine-2,3-dione derivatives. This reaction is based on the cycloaddition of diazopropane, generated in the initial oxidative addition step with iodosylbenzene.

## 2. Results and Discussion

### 2.1. Synthesis of New Spiro-Cyclopropane

**Scheme 1:**
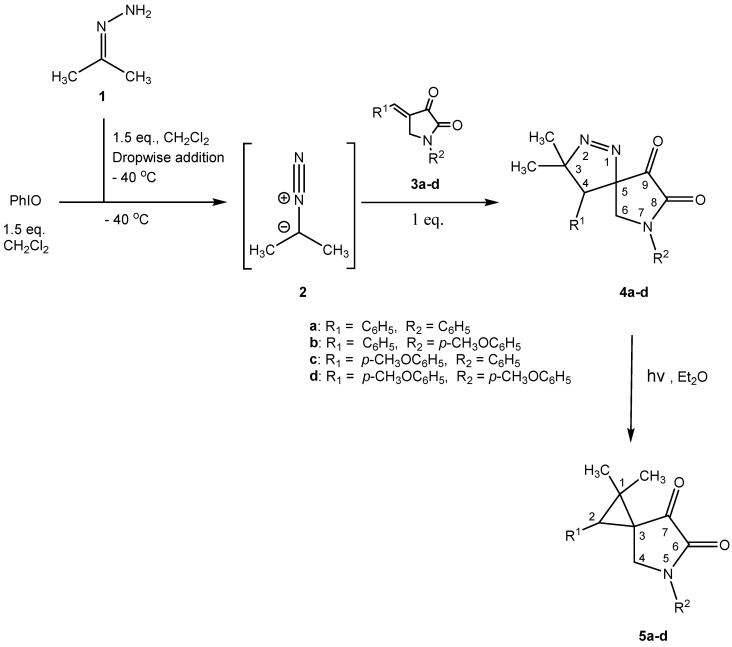
Synthesis and evolution of spiro pyrazole derivatives by cycloaddition of (*E*)-4-arylidene-pyrrolidine-2,3-dione derivatives with 2-diazopropane prepared by oxidizing with iodosylbenzene.

The starting materials **1** was prepared by condensing acetone azine with hydrazine [26], and (*E*)-4-benzylidene-1-phenylpyrrolidine-2,3-dione derivatives **3a–d** were synthesized by the reaction between 1-arylpyrrolidine-2,3-diones and aldehydes, as described in the literature [27,28,29,30]. Diazopropane **2** was prepared in situ by oxidizing acetone azine using iodosylbenzene according to the literature procedure [31]. 1,3-dipolar cycloadditions of 2-diazopropane **2** to (*E*)-4-benzylidene-1-phenylpyrrolidine-2,3-dione derivatives **3a–d** readily occur in dichloromethane at −40 °C. The reaction medium is left stirring for one hour at this temperature, then brought back to 0 °C gradually in a cryostat (Scheme 1) [32].

The 1,3-dipoar cycloaddition of 2-diazopropane is, in each case, regiospecific. The chemical shifts of C5 (99.4 to 99.7 ppm) are in excellent agreement with those usually obtained when this quaternary carbon is attached to the nitrogen atom [25]. The FT-IR spectra of all the synthesized pyrazolines **4a–d** showed absorption bands in the range of 1525–1530 cm^−1^ for the azo group (N=N). The formation of the five-membered ring cycle was confirmed by the presence of two methylene protons for the pyrazoline ring. The chemical shifts of two methylene protons of the compounds **4a–d** were observed between 1.26 and 129 ppm and 1.72–1.79 ppm. One prominent doublet δ = 2.90–3.25 ppm, *J* = 18.3 Hz appeared in the ^1^H-NMR spectra of pyrazolines **4a–d**, which corresponds to the hydrogen atoms H-9. The chemical shifts of the singlet corresponding to the hydrogen atom H-4 appeared at 3.54–3.59 ppm. The chemical shifts of the aromatic protons were found between 6.87 and 7.50 ppm. The ^13^C-NMR of pyrazolines **4a–d** revealed carbonyl carbon peaks at 114.2–160.1 ppm. Aromatic carbon atom chemical shifts appeared in the range of 73.1–200.9 ppm. Under stationary irradiations, pyrazolines **4a–d** yield the discount cyclopropanes **5a–d**. Pyrazoline solutions contained in Pyrex reactors were irradiated with a high-pressure mercury lamp (125 W). The formation of spiro-cyclopropanes **5a–d** was confirmed by ^13^C-NMR. The C-3 spiranic carbon chemical shifts of compounds **5a–d** were observed between 34.0 and 34.3 ppm, confirming that the carbon is not attached to the nitrogen atom. In all cases, we used benzophenone as a sensitizer and dichloromethane as the solvent. Yields were excellent for the breakdown of all pyrazolines (Scheme 1) [25,31].

### 2.2. Biological Activity

The preliminary screening is shown in Table 1. As presented, a number of four spiro-cyclopropanes derivatives were tested against *Aedes aegypti* using acetone as solvent at different concentrations (0.01, 0.005 and 0.001%) and compared to *trans*-chrysanthemic acid and pyrethrin. Cyclopropane derivatives **5a–d** exhibit a knockdown and killing effect similar to or even superior to that of *trans*-chrysanthemic acid and pyrethrin (Table 1). 

Table 2 summarizes the activity results of cyclopropane derivatives **5a–c** against *Aedes aegypti* in the vapor phase. As revealed, cyclopropane **5c** exhibits higher activity than *trans*-chrysanthemic acid and pyrethrin. In fact, the achievement of T90 using *trans*-chrysanthemic acid and pyrethrin required, respectively, 40 min and 38 min, while it just needed 24 min using cyclopropane **5c**. 

Cyclopropanes **5a–d** conferred a complete knockdown after 8 h and mortality after 24 h against *Aedes aegypti*. Additionally, the biological results registered for cyclopropanes **5b–d** (Table 2) showed a slower knockdown in terms of K10, K50 and T90. In order to estimate the insecticidal activity of cyclopropane **5a–d** as space spray against *Aedes aegypti* and Musca domestica, supplementary evaluations were conducted, and the results are shown in Table 3. All cyclopropane derivatives conferred complete knockdown against *Aedes aegypti* within 1 h and mortality after 24 h. Particularly, cyclopropane **5c** showed a better knockdown effect than that of *trans*-chrysanthemic acid and pyrethrin (Table 3). 

Cyclopropane **5c** achieved the quickest knockdown and the best killing effect against *Musca domestica* (Table 4). 

### 2.3. Docking Studies 

**Figure 2:**
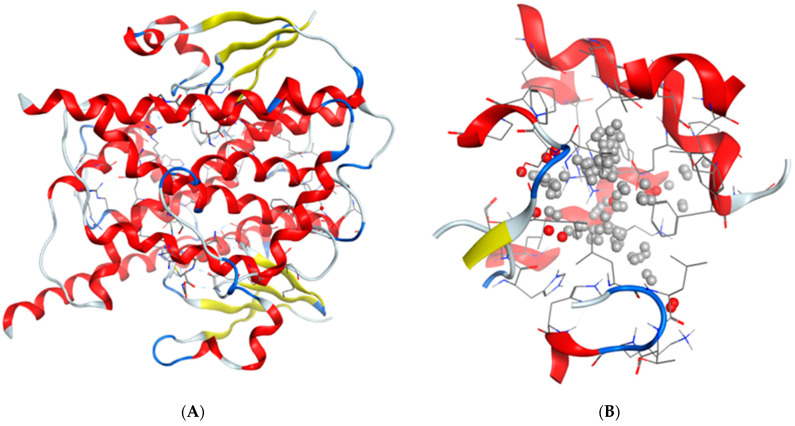
Protein 5FT3 (**A**); largest pocket 5FT3 (**B**).

The objective of this part is the application of molecular docking of spiro-cyclopropane derivatives in order to find possible protein targets in which they present insecticidal activity. We examine how cyclopropanes derivatives might approach the active site of the main protease of mosquitoes (*Aedes aegypti* PDB: 5FT3) (Figure 2). The results showed that all selected inhibitors and drugs were in the pocket of the target proteins. Interaction results were evaluated with the docking score (S). The scoring function is used to predict the binding affinity of both ligand and target once it is docked. Inhibitors with the lowest S score tend to establish a strong interaction with proteins. The docking scores for the drugs approved antiviral activity against 5FT3 proteases were calculated using MOE, applying the same parameters as those used for computing the docking scores for the **5a–d** against 5FT3 proteases in this study.

**Figure 3:**
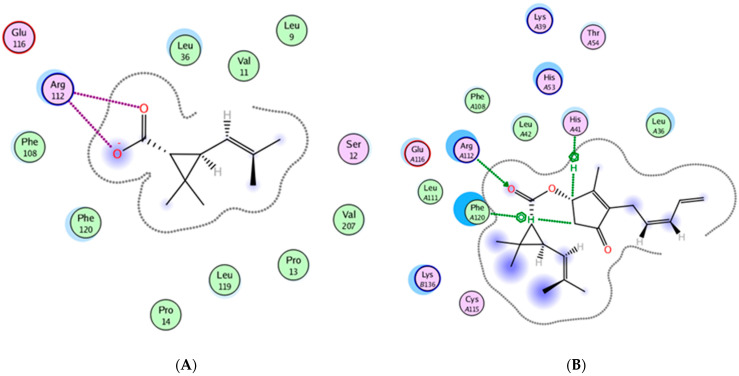
Two-dimensional interaction images of docking conformations of *trans*-chrysanthemic acid and pyrethrin with the active proteins: (**A**) interaction *trans*-chrysanthemic acid and protein 5FT3; (**B**) interactions pyrethrin and protein 5FT3.

*Trans*-chrysanthemic acid and pyrethrin proved to be very attractive thanks to the small doses needed to kill insects. Both *trans*-chrysanthemic acid and pyrethrin exhibited a good interaction with protein 5FT3. The docking of *trans*-chrysanthemic acid with protein 5FT3 shows two polar interactions of the oxygens of carboxylic acid with the Arg112 active amino acid residue (Figure 3A). The pyrethrin docking with protein 5FT3 presents two arene-H interactions of cyclopentane with the His41 and Phe120 active amino acid residues. In this case, the docking score was −6.6755 kcal/mol (Figure 3B).

**Figure 4:**
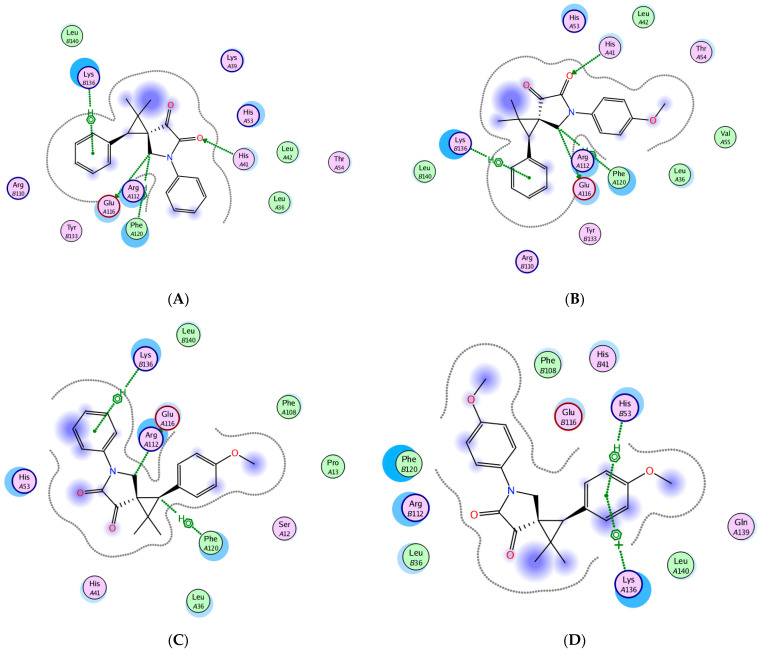
Two-dimensional interaction images of docking conformations of the cyclopropanes **5a-d** with the active protein 5FT3. (**A**) Interaction cyclopropane **5a** and protein 5FT3, (**B**) Interactions cyclopropane **5b** and protein 5FT3, (**C**) Interaction cyclopropane **5c** and protein 5FT3, (**D**) Interaction cyclopropane **5d** and protein 5FT3.

A reasonable comparative study was conducted between the spiro-cyclopropane derivatives **5a–d**, *trans*-chrysanthemic acid and pyrethrin. Docking scores were detected for **5a–d**, ranging from −5.1607 kcal/mol to −6.1161 kcal/mol. The most potent ligand, cyclopropane **5c** docking with protein 5FT3, formed one polar interaction with the Arg112 active amino acid residue. Lys136 and Phe120 exhibited arene-H bonds with the proton of the benzene group and cyclopropane ring, respectively. In this case, the docking score was −6.1161 kcal/mol (Figure 4).

**Figure 5:**
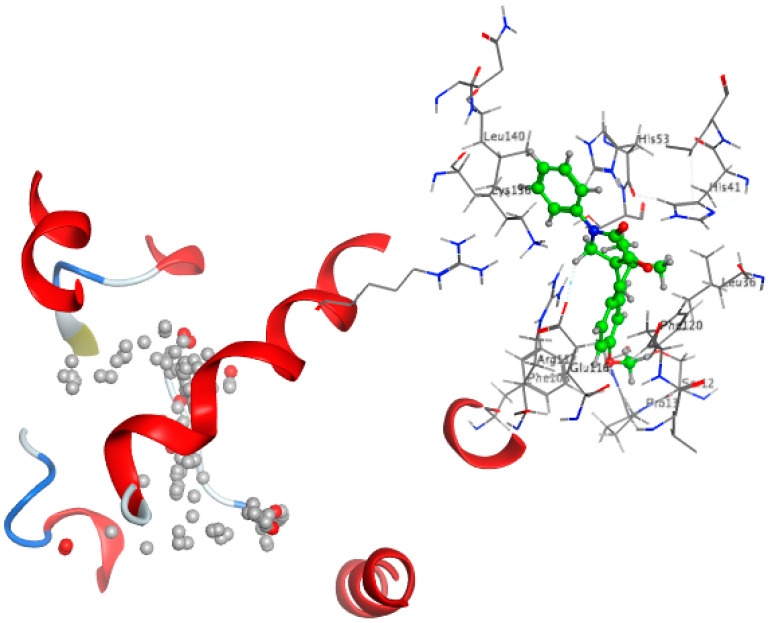
The molecular docking of cyclopropane **5c** against protein 5FT3.

The 3D image of the molecular docking of cyclopropane **5c** against protein 5ft3 is shown in Figure 5. Cyclopropanes **5a** and **5b** formed three hydrogens bonds with the Glu116, Phe120 and His41 active amino acid residues and one H-arene interaction with His4. Cyclopropane **5d** formed two H-arene interactions with His53 and Lys136 active amino acid residues.

## 3. Conclusions

In summary, we have established an efficient, facile and safe batch preparation of *gem*-dimethyl spiro-cyclopropanes through the photodenitrogenation of spiro-pyrazolines obtained by 1,3-dipolar cycloaddition of highly unstable 2-diazopropane starting from acetone hydrazone and using iodosylbenzene as a perfect oxidizing agent. The reaction of 2-diazopropane with (*E*)-4-benzylidene-1-phenylpyrrolidine-2,3-dione derivatives **3a–d** is regiospecific. The presented results demonstrate an excellent insecticidal activity of cyclopropane **5c** against mosquitoes (*Aedes aegypti*) and house flies (*Musca domestica*). This result is confirmed by virtual screening established using molecular docking, and we believe that the sipro-cyclopropane derivatives could aid in insecticidal drug discovery. This is clearly manifested in the bond affinity obtained for cyclopropane **5c,** indicating good binding with the important active amino acid residue of the 5FT3 protein.

## 4. Experimental Section

### 4.1. General Information 

Commercially obtainable reagents were used as-supplied or purified by standard methods where required. Non-commercial starting materials were synthesized according to literature procedures. All (*E*)-4-benzylidene-1-phenylpyrrolidine-2,3-dione derivatives and acetone hydrazone preparation, along with cyclopropanation reactions, were run under a nitrogen atmosphere with oven-dried glassware by means of the usual methods for manipulating air-sensitive product. Dry dichloromethane was prepared by filtration under nitrogen concluded an alumina drying column on a purification system. Thin-layer chromatography was performed on silica gel 254 plates (Merck) with UV (254 nm) visualization, whereas chromatographic separations were conducted on silica gel Si-60–7734. IR spectra (KBr) were recorded on an FTIR 5300 spectrometer. Electrospray Ionization (E.S.I.) mass spectra were measured on a Bruker MicroToF 2. NMR spectra were obtained on a Bruker AV 300 spectrometer operating at 300 MHz for ^1^H and at 75.47 MHz for ^13^C. Melting points were determined on a Buchi-510 capillary melting point apparatus. The coupling constants J are given in Hertz. The spectra were recorded in CDCl_3_ as solvent at room temperature. Elemental analysis was recorded on a PERKIN–ELMER 240B microanalyzer.

### 4.2. Synthesis of Iodosylbenzene

Iodosylbenzene diacetate (3.0 g, 9.2 mmol, 1.0 equiv) was located in a 100 mL round-bottom flask, and 55 mL of NaOH (3M, 165 mmol, 18.0 equiv) was added. The reaction mixture was stirred for 1 h. The crude product was filtered using a funnel and rinsed with water, and the residual product was then abundantly rinsed with chloroform (200 mL). After dried under reduced pressure immediate, the solid was grounded and place back under vacuum for an additional 1 h. The iodosylbenzene was therefore isolated as a yellow solid (1.6 g, 80%) [28]. 

### 4.3. Synthesis of 1,2,7-Triazaspiro[4.4]non-1-ene-8,9-Dione Derivatives (***4a–d***)

In a round-bottom, a threaded inlet 25 mL glass vial equipped with a magnetic bar stirrer was balanced iodosylbenzene (400.0 mg, 1.80 mmol, 1.5 equiv). The bottom was then covered and blushed with nitrogen for five minutes, after which dichloromethane (6 mL) was added. A freshly distillated 6 mL acetone hydrazone (1.80 mmol, 1.5 equiv) dichloromethane was then added over 60 min. The selected alkenes (1.20 mmol, 1.0 equiv) were added. The reaction mixture was vigorously stirred for an additional 30 min at −40 °C. The resulting mixture was then filtrated over celite^®^ and abundantly rinsed with dichloromethane, trailed by evaporation under reduced pressure. The mixture was dried over anhydrous sodium sulfate, filtered and concentrated. The crude cycloadduct was then purified by silica gel chromatography to give the pyrazolines **4a–d**.

#### 4.3.1. 3,3-Dimethyl-4,7-Diphenyl-1,2,7-Triazaspiro[4.4]non-1-ene-8,9-Dione (**4a**)

Yield (85%), white solid. Mp = 139–140 °C, Rf = 0.27 (cyclohexane/EtOAc 3:7). IR (KBr) ν_max_/cm^−1^: 1530 (N=N). ^1^H NMR (300 MHz, CDCl_3_) δ 1.28 (s, 3H, CH_3_), 1.75 (s, 3H, CH_3_), 2.91 and 3.25 (AA’, H_9_, *J* = 18.3 Hz), 3.58 (s, 1H, H_4_), 6.92–7.49 (m, 10H, H_arom_). ^13^C NMR (75.47 MHz, CDCl_3_) δ 24.2 and 28.3 (CH_3_), 37.5 (C_6_), 52.8 (C_4_), 97.4 (C_3_), 99.4 (C_5_), 126.4–135.5 (C_arom_), 173.1 (C_8_), 200.2 (C_9_). HRMS (ESI) Calcd for C_20_H_19_N_3_O_2_ 333.1477 [M^+^]. Found: 333.1480. Elemental analysis: C_20_H_19_N_3_O_2_ requires C, 72.05, H, 5.74, N, 12.60%; found C, 72.01, H, 5.77, N, 12.57%.

#### 4.3.2. 4-(4-Methoxyphenyl)-3,3-Dimethyl-7-Phenyl-1,2,7-Triazaspiro[4.4]non-1-ene-8,9-Dione (**4b**)

Yield (80%), white solid. Mp = 112–113 °C, Rf = 0.27 (cyclohexane/EtOAc 3:7). IR (KBr) ν_max_/cm^−1^: 1525 (N=N). ^1^H NMR (300 MHz, CDCl_3_) δ 1.26 (s, 3H, CH_3_), 1.72 (s, 3H, CH_3_), 2.92 and 3.20 (AA’, H_6_, *J* = 18.3 Hz), 3.54 (s, 1H, H_4_), 3.80 (s, 3H, OCH_3_), 6.87–7.50 (m, 9H, H_arom_). ^13^C NMR (75.47 MHz, CDCl_3_) δ 24.1 and 28.1 (CH_3_), 37.3 (C_6_), 52.1 (C_4_), 97.0 (C_3_), 99.2 (C_5_), 114.2–159.2 (C_arom_), 173.1 (C_8_), 200.3 (C_9_). HRMS (ESI) Calcd for C_21_H_21_N_3_O_3_ 363.1583 [M^+^]. Found: 363.1590. Elemental analysis: C_21_H_21_N_3_O_3_ requires C, 69.41; H, 5.82; N, 11.56%; found C, 69.38; H, 5.88; N, 11.51%.

#### 4.3.3. 7-(4-Methoxyphenyl)-3,3-Dimethyl-4-Phenyl-1,2,7-Triazaspiro[4.4]non-1-ene-8,9-Dione (**4c**)

Yield (75%), white solid. Mp = 123–124 °C, Rf = 0.29 (cyclohexane/EtOAc 3:7). IR (KBr) ν_max_/cm^−1^: 1530 (N=N). ^1^H NMR (300 MHz, CDCl_3_) δ 1.29 (s, 3H, CH_3_), 1.76 (s, 3H, CH_3_), 2.90 and 3.25 (AA’, H_9_, *J* = 18.3 Hz), 3.59 (s, 1H, H_4_), 3.84 (s, 3H, OCH_3_), 6.93–7.37 (m, 9H, H_arom_). ^13^C NMR (CDCl_3_; 75.47 MHz) δ 23.5 and 28,6 (CH_3_), 37.8 (C_6_), 53.1 (C_4_), 55.9 (OCH_3_), 97.7 (C_3_), 99.7 (C_5_), 114.9–160.1 (C_arom_), 173.7 (C_8_) and 200.8 (C_9_). HRMS (ESI) Calcd for C_21_H_21_N_3_O_3_ 363.1583 [M^+^]. Found: 363.1580. Elemental analysis: C_21_H_21_N_3_O_3_ requires C, 69.41; H, 5.82; N, 11.56%; found C, 69.39; H, 5.85; N, 11.52%.

#### 4.3.4. 4,7-bis(4-Methoxyphenyl)-3,3-Dimethyl-1,2,7-Triazaspiro[4.4]non-1-ene-8,9-Dione (**4d**)

Yield (65%), white solid. Mp = 97–98 °C, Rf = 0.26 (cyclohexane/EtOAc 3:7). IR (KBr) ν_max_/cm^−1^: 1530 (N=N). ^1^H NMR (300 MHz, CDCl_3_) δ 1.27 (s, 3H, CH_3_), 1.74 (s, 3H, CH_3_), 2.92 and 3.21 (AA’, H_9_, *J* = 18.3 Hz), 3.55 (s, 1H, H_4_), 3.83 (s, 3H, OCH_3_), 3.82 (s, 3H, OCH_3_), 6.87–7.28 (m, 8H, H_arom_). ^13^C NMR (CDCl_3_; 75.47 MHz) δ 24.5 and 28.5 (CH_3_), 37.6 (C_6_), 52.5 (C_4_), 55.7 (OCH_3_), 55.9 (OCH_3_), 97.3 (C_3_), 99.4 (C_5_), 114.5–160.1 (C_arom_), 173.6 (C_8_) and 200.9 (C_9_). HRMS (ESI) Calcd for C_22_H_23_N_3_O_4_ 393.1689 [M^+^]. Found: 393.1685. Elemental analysis: C_22_H_23_N_3_O_4_ requires C, 67.16; H, 5.89; N, 10.68%; found C, 67.19; H, 5.91; N, 10.64%.

### 4.4. General Procedure for the Irradiation of the Spiro-Pyrazolines (***4a,b***)

All irradiations were carried out using similar conditions. Five moles of pyrazoline were dissolved in ether pretreated by stirring with solid (NaCO_3_), filtered and flushed with nitrogen and irradiated at 5 °C for a total of 30 min or until the starting material was consumed (TLC). After this period, the solvent was removed in a vacuum without heating to give a brown oil, which was subjected to rapid silica filtration. Recrystallization from dichloromethane/light petroleum.

#### 4.4.1. 1,1-Dimethyl-2,5-Diphenyl-5-Azaspiro[2.4]heptane-6,7-Dione (**5a**)

Yield (85%), white solid. Mp = 186–187 °C, Rf = 0.32 (cyclohexane/EtOAc 3:7). ^1^H NMR (300 MHz, CDCl_3_) δ 1.20 (s, 3H, CH_3_), 1.64 (s, 3H, CH_3_), 2.59 and 2.74 (AA’, H_4_, *J* = 19.2 Hz), 3.04 (s, 1H, H_2_), 7.14–7.53 (m, 10H, H_arom_). ^13^C NMR (CDCl_3_; 75.47 MHz) δ 20.7 and 20.9 (CH_3_), 31.3 (C_4_), 31.9 (C_1_), 34.1 (C_3_), 40.3 (C_2_), 126.7–134.3 (C_arom_), 175.2 (C_6_) 200.5 (C_7_). HRMS (ESI) Calcd for C_20_H_19_NO_2_ 305.1416 [M^+^]. Found: 305.1410. Elemental analysis: C_20_H_19_NO_2_ requires C, 78.66; H, 6.27; N, 4.59%; found C, 78.68; H, 6.25; N, 4.55%.

#### 4.4.2. 5-(4-Methoxyphenyl)-1,1-Dimethyl-2-Phenyl-5-Azaspiro[2.4]heptane-6,7-Dione (**5b**)

Yield (90%), white solid. Mp = 145–146 °C, Rf = 0.31 (cyclohexane/EtOAc 3:7). ^1^H NMR (300 MHz, CDCl_3_) δ 1.12 (s, 3H, CH_3_), 1.64 (s, 3H, CH_3_), 2.59 and 2.74 (AA’, H_7_4 *J* = 19.2 Hz), 3.04 (s, 1H, H_2_), 3.85 (s, 3H, OCH_3_), 7.00–7.40 (m, 9H, H_arom_). ^13^C NMR (CDCl_3_; 75.47 MHz) δ 21.1 and 21.2 (CH_3_), 31.6 (C_4_), 32.1 (C_1_), 34.3 (C_3_), 40.5 (C_2_), 55.9 (OCH_3_), 114.8–159.8 (C_arom_), 175.9 (C_6_) 200.2 (C_7_). HRMS (ESI) Calcd for C_21_H_21_NO_3_ 335.1521 [M^+^]. Found: 335.1525. Elemental analysis: C_21_H_21_NO_3_ requires C, 75.20; H, 6.31; N, 4.18%; found C, C, 75.18; H, 6.29; N, 4.20%.

#### 4.4.3. 2-(4-Methoxyphenyl)-1,1-Dimethyl-5-Phenyl-5-Azaspiro[2.4]heptane-6,7-Dione (**5c**)

Yield (75%), white solid. Mp = 151–153 °C, Rf = 0.31 (cyclohexane/EtOAc 3:7). ^1^H NMR (300 MHz, CDCl_3_) δ 1.18 (s, 3H, CH_3_), 1.62 (s, 3H, CH_3_), 2.63 and 2.79 (AA’, H_7_4 *J* = 19.2 Hz), 3.03 (s, 1H, H_2_), 3.84 (s, 3H, OCH_3_), 6.99–7.39 (m, 9H, H_arom_). ^13^C NMR (CDCl_3_; 75.47 MHz) δ 20.8 and 20.9 (CH_3_), 31.3 (C_4_), 31.8 (C_1_), 34.0 (C_3_), 40.2 (C_2_), 55.6 (OCH_3_), 114.5–159.4 (C_arom_), 175.6 (C_6_) 200.8 (C_7_). HRMS (ESI) Calcd for C_21_H_21_NO_3_ 335.1521 [M^+^]. Found: 335.1516. Elemental analysis: C_21_H_21_NO_3_ requires C, 75.20; H, 6.31; N, 4.18%; found C, C, 75.17; H, 6.28; N, 4.17%.

#### 4.4.4. 2,5-bis(4-Methoxyphenyl)-1,1-Dimethyl-5-Azaspiro[2.4]heptane-6,7-Dione (**5d**) 

Yield (70%), white solid. Mp = 163–164 °C, Rf = 0.29 (cyclohexane/EtOAc 3:7). ^1^H NMR (300 MHz, CDCl_3_) δ 1.17 (s, 3H, CH_3_), 1.61 (s, 3H, CH_3_), 2.61 and 2.76 (AA’, H_7_4 *J* = 19.2 Hz), 2.95 (s, 1H, H_2_), 3.81 (s, 3H, OCH_3_), 3.84 (s, 3H, OCH_3_), 6.87–8.04 (m, 8H, H_arom_). ^13^C NMR (CDCl_3_; 75.47 MHz) δ 20.7 and 20.9 (CH_3_), 31.3 (C_4_), 31.9 (C_1_), 34.0 (C_3_), 39.7 (C_2_), 55.4 (OCH_3_), 55.6 (OCH_3_), 113.8–159.4 (C_arom_), 175.6 (C_6_) 200.9 (C_7_). HRMS (ESI) Calcd for C_22_H_23_NO_4_ 365.1627 [M^+^]. Found: 365.1630. Elemental analysis: C_22_H_23_NO_4_ requires C, 72.31; H, 6.34; N, 3.83%; found C, 72.29; H, 6.31; N, 3.81%.

### 4.5. Study of the Insecticidal Activity of Spiro-Cyclopropane ***5a–d***, the Trans-Chrysanthemic Acid and Pyrethrin

Biological laboratory tests have been carried out to screen new products for their effectiveness against mosquitoes (*Aedes aegypti*) and house flies (*Musca domestica*). Detailed rearing procedures for each insect are provided in the Supplementary Material.

#### 4.5.1. Effects of Cyclopropanes **5a–d** at Contact Action against *Aedes aegypti*

The mosquitoes were obtained in the egg state and subsequently stored at a temperature of 30 °C and relative humidity of 47%. Each test was performed in a test tube and was repeated 3 times. Acetone was used as the dilution solvent. The spiro-cyclopropanes **5a–d**, the *trans*-chrysanthemic acid and pyrethrin were dissolved in acetone at different concentrations (C = 0.001, 0.005 and 0.01%). The sheets of cellulose paper were immersed with the solutions prepared at the rate of 0.12 μL of mm^−2^ solution and then dried and placed in the test tubes. The tests were carried out with ten female mosquitoes aged 1 to 3 days. The mosquitoes were released inside the tube, and then the cap was closed. Knockdown of *Aedes aegypti* was observed at the indicated intervals up to 60 min. Subsequently, the mosquitoes were placed in untreated glass (250 mL) containing cotton soaked in firm sugar water. Rollover and mortality were observed for 24 h (Table 1).

#### 4.5.2. Effects of Cyclopropanes **5a–d** in the Vapor Phase against *Aedes aegypti*

This test was carried out in a stainless-steel enclosure with three doors, with a volume of 22.5 m^3^ and the following dimensions: length 3 m; width 3 m; height 2.5 m. The temperature inside the chamber was maintained at 21–25 °C and the relative humidity at 50–60%. The mosquitoes were introduced into mesh polyester cages of dimensions: length 8 cm, diameter 8 cm and mesh 2 mm. Then, they were suspended from the ceiling of the stainless-steel enclosure at a height of 1.5 and 0.5 m from the walls (see Figure 1). The insecticidal effect was studied by keeping 20 mixed-sex mosquitoes for 3 days in each wire cage. Cellulose papers (10 × 10 cm) immersed in a solution of each cyclopropane, *trans*-chrysanthemic acid and pyrethrin (5 mg) were placed above the fans for 8 h. Let T10, T50 and T90 represent the times required to kill 10, 50 and 90% of mosquitoes, respectively. Results were recorded every 2 h. The insects were removed from the cages after 12 h and transferred to glasses (250 mL) provided with cellulose pads soaked in a 10% sugar solution. Mortality was recorded 24 h after treatment (Table 2).

#### 4.5.3. Aerosols Effects of Cyclopropanes **5a–d** Liquid Formulation against *Aedes aegypti* and Musca Domestica

This test was carried out in a stainless-steel enclosure with three doors, with a volume of 22.5 m^3^ and the following dimensions: length 3 m; width 3 m; height 2.5 m. The temperature inside the chamber was maintained at 21–25 °C and the relative humidity at 50–60%. The mosquitoes were introduced into mesh polyester cages (length 8 cm, diameter 8 cm, mesh 2 mm) and were suspended from the ceiling of the stainless-steel enclosure at a height of 1.5 and 0.5 m from the walls. The prepared solution was sprayed vertically at a height of 1 m in the center of the enceinte. A fan placed on the floor operated for 5 min. The times T10, T50 and T90 were recorded every 2 h. The insects were removed from the cages after 12 h and transferred to glasses (250 mL) provided with cellulose pads soaked in a 10% sugar solution. Mortality was recorded 24 h after treatment (Table 3 and Table 4).

### 4.6. Docking

#### 4.6.1. Preparation of Cyclopropanes **5a–d** and Trans-Chrysanthemic Acid for Docking Analysis 

All these molecular structures were reproduced in Chem-drawn ultra-version, and then all ligands were saved in mol format, with the aim of opening these files in MOE after structure preparation, and these were protonated 3D with energy minimized through MOE using default parameters. 

#### 4.6.2. Preparation of Protein and Molecular Docking 

The crystal structure of the protein (PDB: 5FT3) was obtained from the RSCB data bank (https://www.rcsb.org, accessed on 8 January 2021). The protein was prepared by removing the complexed inhibitor ligand and water molecules. Then, the polar hydrogens were added, followed by appending Kollman charges. Hence, the grid box with dimensions of 40 × 40 × 40 points, spacing of 1.0 Å and centered with coordinates x: −11.993, y: 15.425, and z: 65.951, was generated based on the N3 binding position in the target protein binding site. While performing docking, the ligand atom was selected, and rescoring1 was set at London dG and rescoring2 at GBVI/WSA dG, running to note the ligand interaction with protein. Protein–ligand docking score, ligand properties and 2D and 3D structures were saved. The docking calculations have been carried out using an Intel (R) Core (TM) CPU @ 3.4 GHz Workstation.

## Figures and Tables

**Table 1:** Effects of cyclopropanes **5a–d**, *trans*-chrysanthemic acid and pyrethrin at contact action against *Aedes aegypti* at three concentrations.

		Number of Mosquitoes that Fell on the Ground				
Products	Concentration	1 min	10 min	30 min	60 min	Number of Dead Mosquitoes after 24 h
Acetone	n/a	8.3	0.3	0	0	0.6
*Trans*-chrysanthemic acid (Reference)	0.001	7.6	8.3	10	10	9.3
	0.005	8	9.3	10	10	10
	0.01	9.3	10	10	10	10
Pyrethrin (Reference)	0.001	8.3	8.9	10	10	10
	0.005	9.3	9.5	10	10	10
	0.01	9.5	10	10	10	10
Cyclopropane **5a**	0.001	7.3	8.3	8.9	10	10
	0.005	7.8	8.5	8.9	10	10
	0.01	8.5	8.9	10	10	10
Cyclopropane **5b**	0.001	7.6	8.3	8.9	10	10
	0.005	8.3	8.9	9.8	10	10
	0.01	8.6	9.3	10	10	10
Cyclopropane **5c**	0.001	8.9	9.5	10	10	10
	0.005	9.3	10	10	10	10
	0.01	9.8	10	10	10	10
Cyclopropane **5d**	0.001	7.3	7.6	8.3	9.3	10
	0.005	7.6	8.3	8.9	9.8	10
	0.01	8.3	8.9	9.5	10	10

**Table 2:** Effects of cyclopropanes **5a–d** versus *trans*-chrysanthemic acid and pyrethrin in the vapor phase against *Aedes aegypti*.

Products (5 mg per Cellulose Paper)	Insect Exposure (h)	Knockdown Time (min)			Percentage Knockdown (8 h)	Percentage Knockdown (24 h)
		T10	T50	T90		
*Trans*-chrysanthemic acid (reference)	0	23	31	40	100	100
	2	6	10	13	100	100
	4	5	9	12	100	100
Pyrethrin (reference)	0	21	27	38	100	100
	2	5	9	11	100	100
	4	4	8	10	100	100
Cyclopropane **5a**	0	22	33	41	100	100
	2	14	23	33	100	100
	4	12	19	21	100	100
Cyclopropane **5b**	0	22	32	40	100	100
	2	15	22	34	100	100
	4	12	18	19	100	100
Cyclopropane **5c**	0	19	21	24	100	100
	2	4	8	10	100	100
	4	3	7	9	100	100
Cyclopropane **5d**	0	25	36	45	100	100
	2	16	26	35	100	100
	4	14	21	26	100	100

**Table 3:** Biological efficacy of cyclopropanes **5a–d** versus trans-chrysanthemic acid and pyrethrin as a space spray in 22.5 m^3^ chambers against *Aedes aegypti*.

Products (0.05 mg m^−3^)	Knockdown Time (min)			Percentage Knockdown (1 h)	Percentage Knockdown (24 h)
	T10	T50	T90		
*Trans*-chrysanthemic acid	0 min 46 s	1 min 15 s	1 min 37 s	100	100
Pyrethrin	0 min 40 s	1 min 10 s	1 min 25 s	100	100
Cyclopropane **5a**	0 min 54 s	1 min 40 s	2 min 35 s	100	100
Cyclopropane **5b**	1 min 02 s	1 min 55 s	2 min 44 s	100	100
Cyclopropane **5c**	0 min 37 s	1 min 00 s	1 min 20 s	100	100
Cyclopropane **5d**	1 min 20 s	1 min 50 s	3 min 07 s	100	99

**Table 4:** The biological efficacy of cyclopropanes **5a–d** versus trans-chrysanthemic acid and pyrethrin as a space spray in 22.5 m^3^ chambers against *Musca domestica*.

Products (0.05 mg m^−3^)	Knockdown Time (min)			Percentage Knockdown (1 h)	Percentage Knockdown (24 h)
	T10	T50	T90		
*Trans*-chrysanthemic acid	5 min 36 s	7 min 20 s	10 min 07 s	100	78
Pyrethrin	5 min 13 s	6 min 30 s	8 min 09 s	100	96
Cyclopropane **5a**	0 min 54 s	1 min 40 s	2 min 35 s	100	61
Cyclopropane **5b**	1 min 02 s	1 min 55 s	2 min 44 s	100	63
Cyclopropane **5c**	0 min 37 s	1 min 00 s	1 min 20 s	100	100
Cyclopropane **5d**	1 min 20 s	1 min 50 s	3 min 07 s	100	54

## Data Availability

Not applicable.

## Supplementary Materials

The following supporting information can be downloaded at: https://www.mdpi.com/article/10.3390/molecules27082470/s1, Figure S1: 1H-NMR spectrum of compound **4a** recorded in CDCl3, Figure S2: 13C-NMR spectrum of compound **4a** recorded in CDCl3, Figure S3: HRMS mass spectrum of compound **4a**, Figure S4: 1H-NMR spectrum of compound **4b** recorded in CDCl3, Figure S5: 13C-NMR spectrum of compound **4b** recorded in CDCl3, Figure S6: HRMS mass spectrum of compound **4b**, Figure S7: 1H-NMR spectrum of compound **4c** recorded in CDCl3, Figure S8: 13C-NMR spectrum of compound **4c** recorded in CDCl3, Figure S9: HRMS mass spectrum of compound **4c**, Figure S10: 1H-NMR spectrum of compound **4d** recorded in CDCl3, Figure S11: 13C-NMR spectrum of compound **4d** recorded in CDCl3, Figure S12: HRMS mass spectrum of compound **4d**, Figure S13: 1H-NMR spectrum of compound **5a** recorded in CDCl3, Figure S14: 13C-NMR spectrum of compound **5a** recorded in CDCl3, Figure S15: HRMS mass spectrum of compound **5a**, Figure S16: 1H-NMR spectrum of compound **5b** recorded in CDCl3, Figure S17: 13C-NMR spectrum of compound **5b** recorded in CDCl3, Figure S18: HRMS mass spectrum of compound **5b**, Figure S19: 1H-NMR spectrum of compound **5c** recorded in CDCl3, Figure S20: 13C-NMR spectrum of compound **5c** recorded in CDCl3, Figure S21: HRMS mass spectrum of compound **5c**, Figure S22: 1H-NMR spectrum of compound **5d** recorded in CDCl3, Figure S23: 13C-NMR spectrum of compound **5d** recorded in CDCl3, Figure S24: HRMS mass spectrum of compound **5d**.

## Sample Availability

Samples of the compounds **5a–d** are available from the authors.

## References

[1] Mahapatra A., Prasad T., Sharma T. (2021). Pyrimidine: A Review on Anticancer Activity with Key Emphasis on SAR. Future J. Pharm. Sci..

[2] Salaün J., Baird M.S. (1995). Biologically active cyclopropanes and cyclopropenes. Curr. Med. Chem..

[3] Salaün J. (2000). Cyclopropane Derivatives and their Diverse Biological Activities. Top. Curr. Chem..

[4] Donaldson W.A. (2001). Synthesis of Cyclopropane Containing Natural Products. Tetrahedron.

[5] Wessjohann L.A., Brandt W., Thiemann T. (2003). Biosynthesis and Metabolism of Cyclopropane Rings in Natural Compounds. Chem. Rev..

[6] Ben Hamadi N., Msaddek M. (2012). A facile and efficient ultrasound-assisted stereospecific synthesis of novel bicyclo-cyclopropanes. C. R. Chim..

[7] Kulinkovich O.G. (2015). Cyclopropanes in Organic Synthesis.

[8] Huang Q., Larock R.C. (2009). Synthesis of Cyclopropanes by Pd-Catalyzed Activation of Alkyl C−H Bonds. Tetrahedron Lett..

[9] Chung D.S., Lee J.S., Ryu H., Park J., Kim H., Lee J.H., Kim U.B., Lee W.K., Baik M.-H., Lee S.-g. (2018). Palladium-Catalyzed Divergent Cyclopropanation by Regioselective Solvent-Driven C(sp3)−H Bond Activation. Angew. Chem. Int. Ed..

[10] Ben Hamadi N., Lachheb J., Khemiss A. (2003). Cycloaddition dipolaire-1,3 du 2-diazopropane sur des maléimides N-substitués. Photochimie des adduits résultants. J. Soc. Chim. Tun..

[11] Clemenceau A., Thesmar P., Gicquel M., Le Flohic A., Baudoin O. (2020). Direct Synthesis of Cyclopropanes from gem-Dialkyl Groups through Double C–H Activation. J. Am. Chem. Soc..

[12] Gnad F., Reiser O. (2003). Synthesis and Applications of β-Aminocarboxylic Acids Containing a Cyclopropane Ring. Chem. Rev..

[13] Brackmann F., de Meijere A. (2007). Natural Occurrence, Syntheses, and Applications of Cyclopropyl-Group-Containing α-Amino Acids. 1. 1-Aminocyclopropanecarboxylic Acid and Other 2,3-Methanoamino Acids. Chem. Rev..

[14] Krief A. (1994). Synthesis of pyrethroic acids from natural products and from isomeric compounds: Strategy and practice. Pestic. Sci..

[15] Fujitani Y. (1909). Beiträge zur Chemie und Pharmakologie des Insektenpulvers. Arch. Exp. Pathol. Pharmakol..

[16] Gauthier L., Olivier C., Janine C., Christophe M. (2021). Radical Addition of SF5Cl to Cyclopropenes: Synthesis of (Pentafluorosulfanyl)cyclopropanes. Org. Lett..

[17] Alexander J.B., James A. (2021). Stereoselective synthesis and applications of spirocyclic oxindoles. Org. Chem. Front..

[18] Zheng Y., Tice C.M., Singh S.B. (2014). The use of spirocyclic scaffolds in drug discovery. Bioorg. Med. Chem. Lett..

[19] Shichuang M., Weiqi J., Qi L., Tian L., Wenjun W., Hangyu B., Baojun S. (2021). Design, Synthesis, and Study of the Insecticidal Activity of Novel Steroidal 1,3,4-Oxadiazoles. J. Agric. Food Chem..

[20] Sally M.B., Sarah A., Cage A.T. (2005). Proudfoot and J. Allister Vale. Poisoning due to Pyrethroids. Toxicol. Rev..

[21] Elena M., Ona I., José A.C., Ángel Á.L., José L.B., Vicenç B., Rosa M.O. (2003). Photolysis of Chiral 1-Pyrazolines to Cyclopropanes:  Mechanism and Stereospecificity. J. Org. Chem..

[22] Louhichi N., Haouas A., Ben Hamadi N., Msaddek M. (2011). Synthesis and chemistry of pyrazolines derived from diphenyldiazomethane. Heterocycl. Commun..

[23] Hajlaoui K., Guesmi A., Ben Hamadi N., Msaddek M. (2018). Synthesis of Novel Pyrazole–sucrose Derivatives by 1,3-dipolar Cycloaddition. J. Heterocycl. Chem..

[24] Ben Hamadi N., Msaddek M. (2011). The Swern Oxidation: First example of direct oxidation of 2-pyrazolines with “activated” DMSO. C. R. Chim..

[25] Ben Hamadi N., Louhichi N., Msaddek M. (2007). Synthesis and photolysis of hexahydropyrrolo[3,4-c]pyrazole derivatives. J. Chem. Res..

[26] Ben Hamadi N., Msaddek M. (2007). Regio- and Stereoselectivity in 1,3-Dipolar Cycloaddition Reaction of 2-Diazopropane with Benzylidene-N-arylsuccinimide and Benzylidene-N-arylmethylsuccinimide Derivatives: Synthesis of gem-Dimethylcyclopropane. J. Chem. Res..

[27] Day A.C., Whiting M.C. (1970). Acetone hydrazone. Org. Synth..

[28] Sundberg R.J., Pearce B.C., Laurino J.P. (1986). Pyrrolidine-2,3-dione, 1-allylpyrrolidine-2,3-dione and 1-ethoxypyrrolidine-2,3-dione. J. Heterocycl. Chem..

[29] Southwick P.L., Crouch R.T. (1953). The Condensation of Oxalic Esters with Esters of β-Alanine and *N*-Substituted β-Aminopropionic Acids. Synthesis of Some Derivatives of 2,3 Dioxopyrrolidine and 2-Oxo-3-methoxy-3-pyrroline. J. Am. Chem. Soc..

[30] Southwick P.L., Barnas E.F. (1962). Unsaturated Cyclic Sulfones. V. 3-Methyl-2,3-dihydrothiophene 1,1-Dioxide. J. Org. Chem..

[31] Emmanuelle M.D.A., André B.C. (2019). Non-stabilized diazoalkane synthesis via the oxidation of free hydrazones by iodosylbenzene and application in in situ MIRC cyclopropanation. Chem. Sci..

[32] Louhichi N., Houas A., Ben Hamadi N., Msaddek M. (2012). Synthesis and chemistry of new spiro-Δ1-pyrazoline. J. Heterocycl. Chem..

