# Supplementary material for: Synthesis of New Spiro-Cyclopropanes Prepared by Non-Stabilized Diazoalkane Exhibiting an Extremely High Insecticidal Activity
Source: Molecules. 2022 Apr 12;27(8):2470. doi: 10.3390/molecules27082470 (PMC9025669; doi:10.3390/molecules27082470)

Supporting Information

Synthesis of New Spiro-Cyclopropanes Prepared by Non-stabilized Diazoalkane Exhibiting an Extremely High Insecticidal Activity

Naoufel Ben Hamadi [1,2,*] and Ahlem Guesmi [1,2,3]

[1] Chemistry Department, College of Science, IMSIU (Imam Mohammad Ibn Saud Islamic University), P.O. Box 5701, Riyadh 11432, Saudi Arabia.
[2] Laboratory of Heterocyclic Chemistry, Natural Products and Reactivity (LR11ES39), Faculty of Science of Monastir, University of Monastir, Avenue of Environment, 5019 Monastir, Tunisia.
[3] Textile Engineering Laboratory, Higher Institute of Technological Studies of Ksar Hellal, UM (University of Monastir), Tunisia.
Correspondence: nabenhamadi@imamu.edu.sa

Table of Contents

1. Breeding methods

	Page No
**1. Breeding methods**	2
**1.1. Breeding Technique *Aedes aegypti***	2
**1.2. Breeding method *Musca domestica***	3

2. ^{1}H NMR, ^{13}C NMR, and Mass (HRMS) spectroscopy data of pyrazolines and cyclopropanes 4 and 5

	Page No
**Figure S1**: ^{1}H-NMR spectrum of compound **4a** recorded in $CDCl_3$.	4
**Figure S2**: ^{13}C-NMR spectrum of compound **4a** recorded in $CDCl_3$.	5
**Figure S3**: HRMS mass spectrum of compound **4a**.	5
**Figure S4**: ^{1}H-NMR spectrum of compound **4b** recorded in $CDCl_3$.	6
**Figure S5**: ^{13}C-NMR spectrum of compound **4b** recorded in $CDCl_3$.	7
**Figure S6**: HRMS mass spectrum of compound **4b**.	7

Figure S7: ^{1}H-NMR spectrum of compound **4c** recorded in $CDCl_3$. **8**

9

Figure S8: ^{13}C-NMR spectrum of compound **4c** recorded in $CDCl_3$.

9

Figure S9: HRMS mass spectrum of compound **4c**. **10**

Figure S10: ^{1}H-NMR spectrum of compound **4d** recorded in $CDCl_3$.

11

Figure S11: ^{13}C-NMR spectrum of compound **4d** recorded in $CDCl_3$.

11

Figure S12: HRMS mass spectrum of compound **4d**.

12

Figure S13: ^{1}H-NMR spectrum of compound **5a** recorded in $CDCl_3$.

13

Figure S14: ^{13}C-NMR spectrum of compound **5a** recorded in $CDCl_3$.

13

Figure S15: HRMS mass spectrum of compound **5a**.

14

Figure S16: ^{1}H-NMR spectrum of compound **5b** recorded in $CDCl_3$.

15

Figure S17: ^{13}C-NMR spectrum of compound **5b** recorded in $CDCl_3$.

15

Figure S18: HRMS mass spectrum of compound **5b**.

16

Figure S19: ^{1}H-NMR spectrum of compound **5c** recorded in $CDCl_3$.

17

Figure S20: ^{13}C-NMR spectrum of compound **5c** recorded in $CDCl_3$.

17

Figure S21: HRMS mass spectrum of compound **5c**.

18

Figure S22: ^{1}H-NMR spectrum of compound **5d** recorded in $CDCl_3$.

19

Figure S23: ^{13}C-NMR spectrum of compound **5d** recorded in $CDCl_3$.

19

Figure S24: HRMS mass spectrum of compound **5d**.

1. Breeding procedures

1.1. Breeding Technique *Aedes aegypti*

Mosquito eggs are generally provided on filter paper. After classification in they are deposited at precise temperature room (a) until complete to be reared into adults. The species typically controls the length of time that they can be kept for *Aedes aegypti* can be desiccated and deposited for a couple of months. When prepared to be reared, the eggs are located in a bottle full of either deionized water or standup tap water. A tank with standing tap water is usually kept in room (a) for this purpose. The number of eggs located in water depends on the flask dimensions, as they

shouldn't be overloaded. Classically, a flask approximately 15 cm x 30 cm x 10 cm should comprise 100 - 150 eggs. The eggs usually hatch in a limited hours and the larvae are providing with brewer's yeast tablets as a food source. Naturally, 0.25 tablet per day will fodder 100 -150 larvae. Care must be occupied to guarantee that larvae are not swollen. The flasks are checked daily to guarantee that they have sufficient food. No additional alimentation is mandatory for weekends. If a tablet is still existing in the water, no extra one is supplementary until the primary one has vanished.

Once the larvae start emergent into cocoons, the flasks are located into a metal-framed mesh enclosed cage. The adults that develop in the cage are fed on sugar water and castoff as obligatory. The flasks with the grubs and pupae in the cage are still fed as pronounced above. Any flasks in which all the larvae have established and pupae have appeared into adults, or with unwanted larvae should be ice-covered for at smallest 24 hours, to confirm that all insects are dead.

1.2. Breeding method *Musca domestica*

The adults are reserved in cages (45 x 45 x 35 cm) with mesh on the sides and upper part under 12 hours: 12 hours light: dark regime at 25°C ±2°C. Two glasses comprising cellulose, soaked with water as drinking foundation, two glasses with nourishment (sugar and milk powder, 4:1) and two plates, each with about 1500 pupae, are located in the enclosure. Four days later all flies are marked. Five days after marking the females start egg laying on humidified peat, covered with dog food and white cheese in a plastic dish. Two days later the dish is detached. The eggs and hatched larvae were moved into a soup tureen occupied with about 100 g of white cheese mixed with dog food and enclosed with dry fertilizer. The tureen is located on a heater mat for two days. Then, when the additional larval phase has been attained, half of the contented of the casserole is moved into a flask, which is organized as shadows: the floor is protected with about two kg food mixture. The grubs are additional and will be totally enclosed with a 2 cm layer of dry fertilizer. The flask is given into a superior one (diameter 30 cm) containing Vermeculite®. After s sevn days, when the third phase has been accomplished, half a liter of water is supplementary to the medium. By that the caterpillars start creeping into the bigger flask and one day later the minor flask is removed. When all larvae are pupated, they are detached from the Vermeculite by sieving. They are transported into a refrigerator (10°C ± 2°C) to be deposited up to 10 days. After sensational them to area temperature (about 24°C) the adult flies hatch within five days.

2. ^{1}H NMR, ^{13}C NMR, and Mass (HRMS) spectroscopy data of pyrazolines and cyclopropanes 4 and 5

3,3-dimethyl-4,7-diphenyl-1,2,7-triazaspiro[4.4]non-1-ene-8,9-dione (4a)

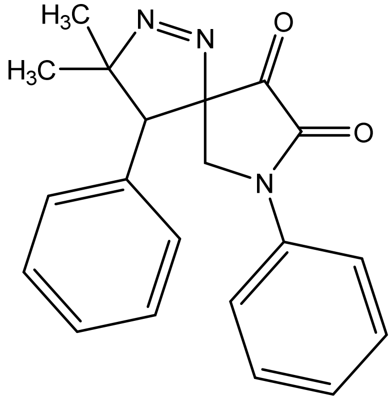

4a

Figure S1: ^{1}H-NMR spectrum of compound **4a** recorded in $CDCl_3$.

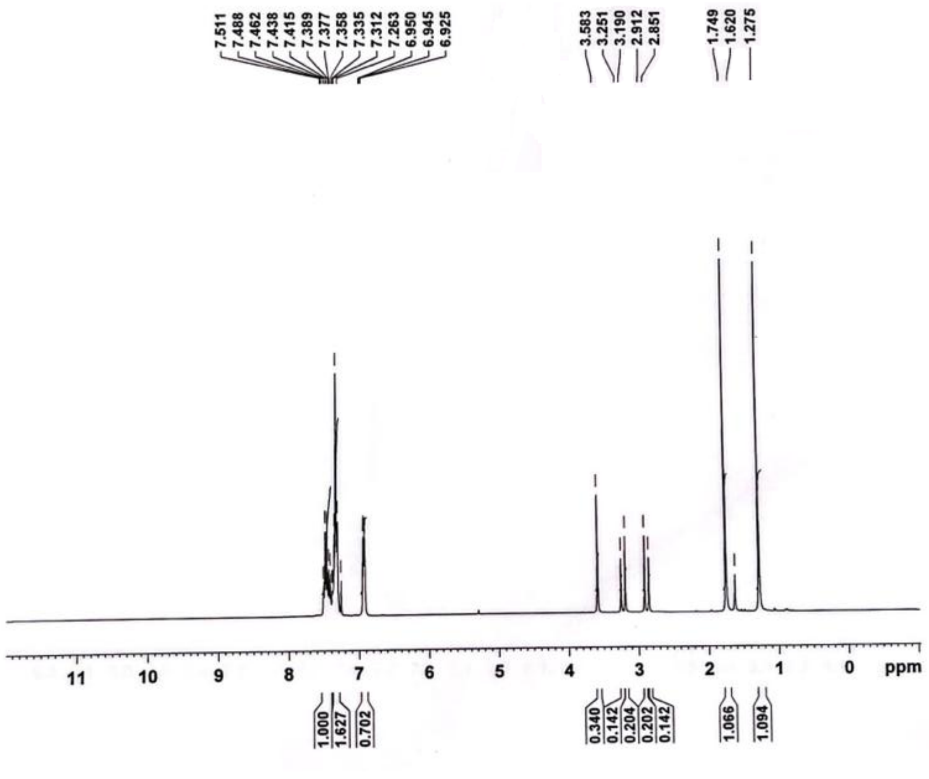

Figure S2: ^{13}C-NMR spectrum of compound **4a** recorded in $CDCl_3$.

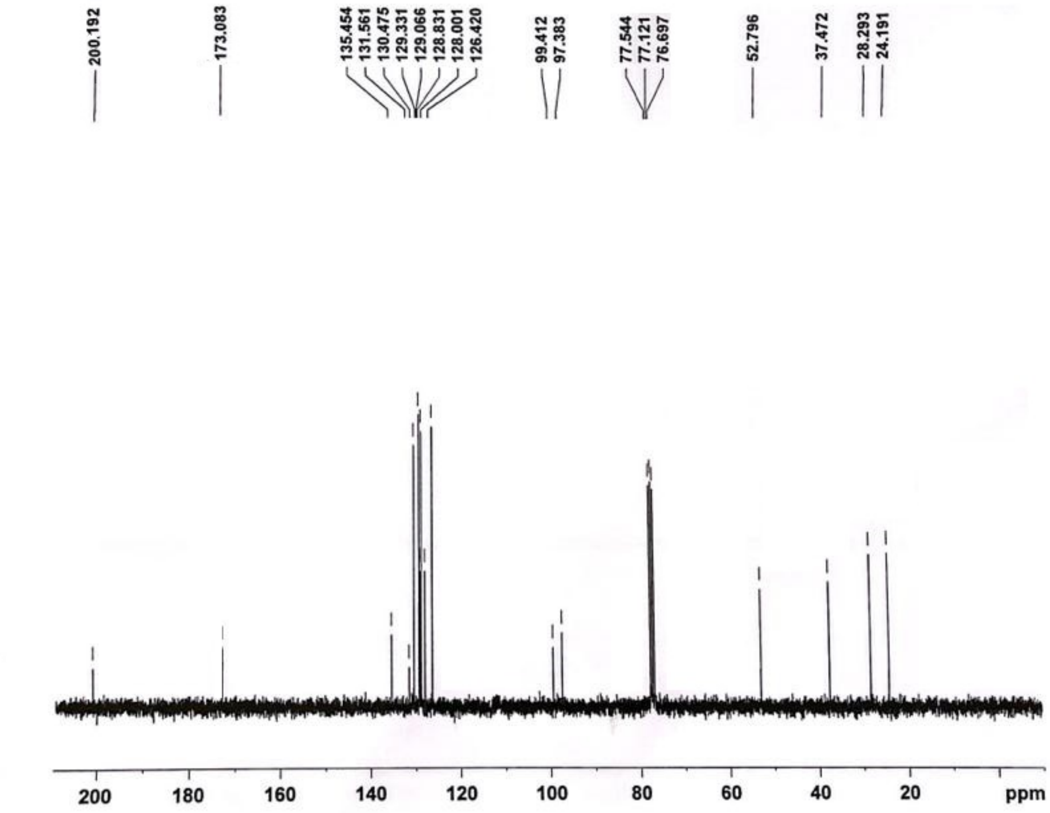

Figure S3: HRMS mass spectrum of compound **4a**.

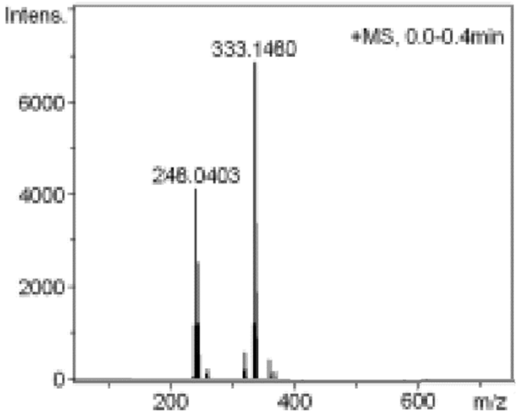

4-(4-methoxyphenyl)-3,3-dimethyl-7-phenyl-1,2,7-triazaspiro[4.4]non-1-ene-8,9-dione (4b)

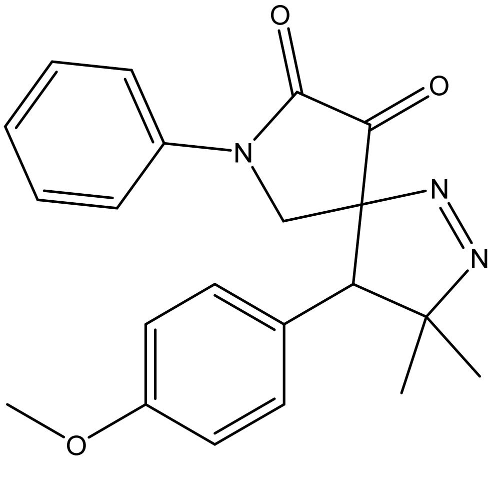

4b

Figure S4: ^{1}H-NMR spectrum of compound **4b** recorded in $CDCl_3$.

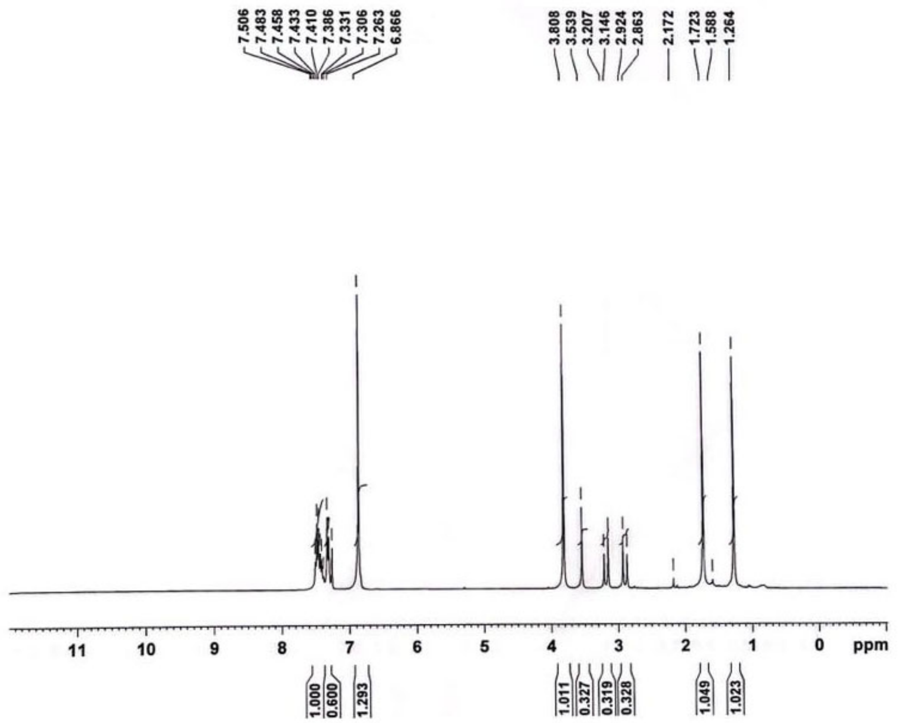

Figure S5: ^{13}C-NMR spectrum of compound **4b** recorded in $CDCl_3$.

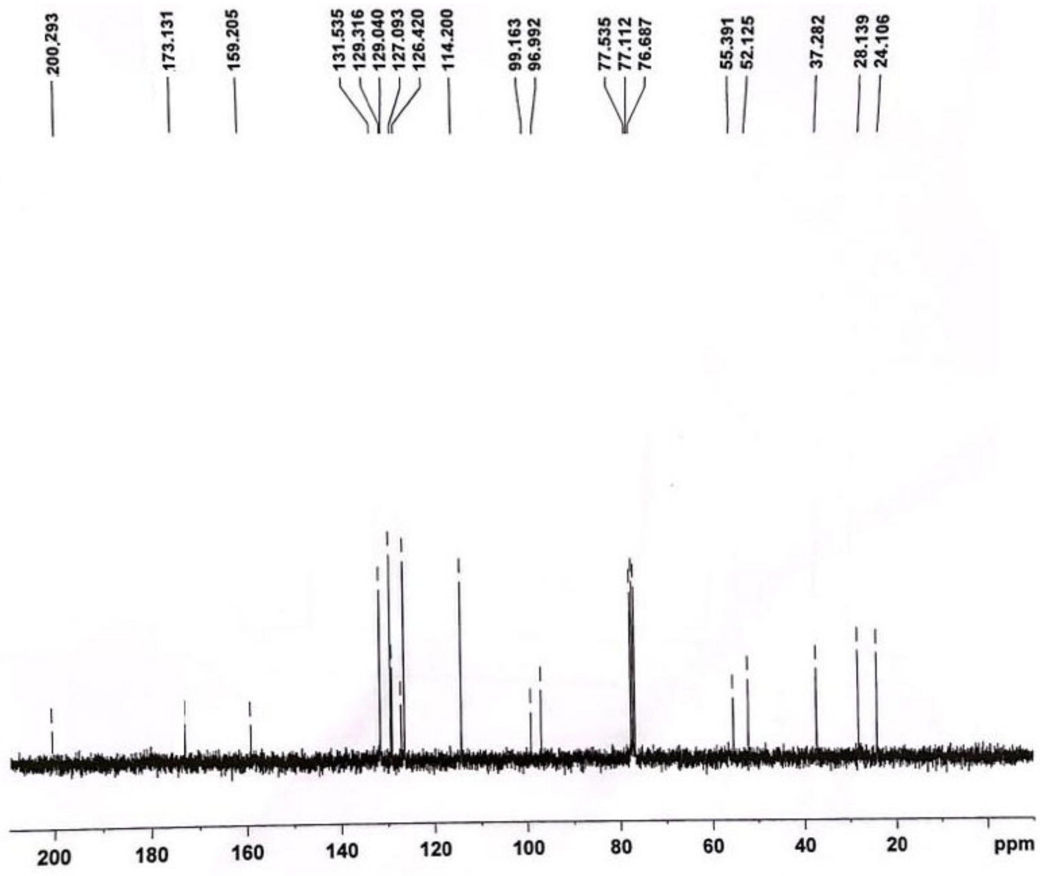

Figure S6: HRMS mass spectrum of compound **4b**.

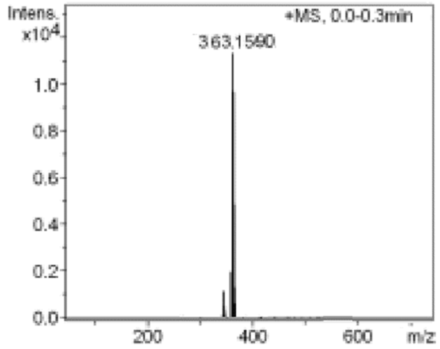

7-(4-methoxyphenyl)-3,3-dimethyl-4-phenyl-1,2,7-triazaspiro[4.4]non-1-ene-8,9-dione (4c)

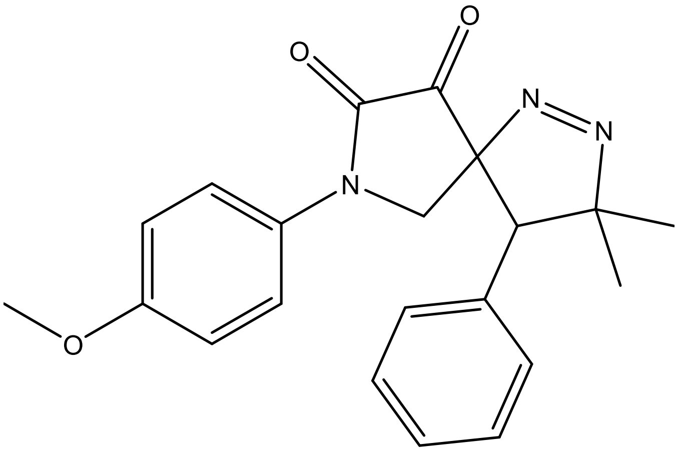

4c

Figure S7: ^{1}H-NMR spectrum of compound **4c** recorded in $CDCl_3$.

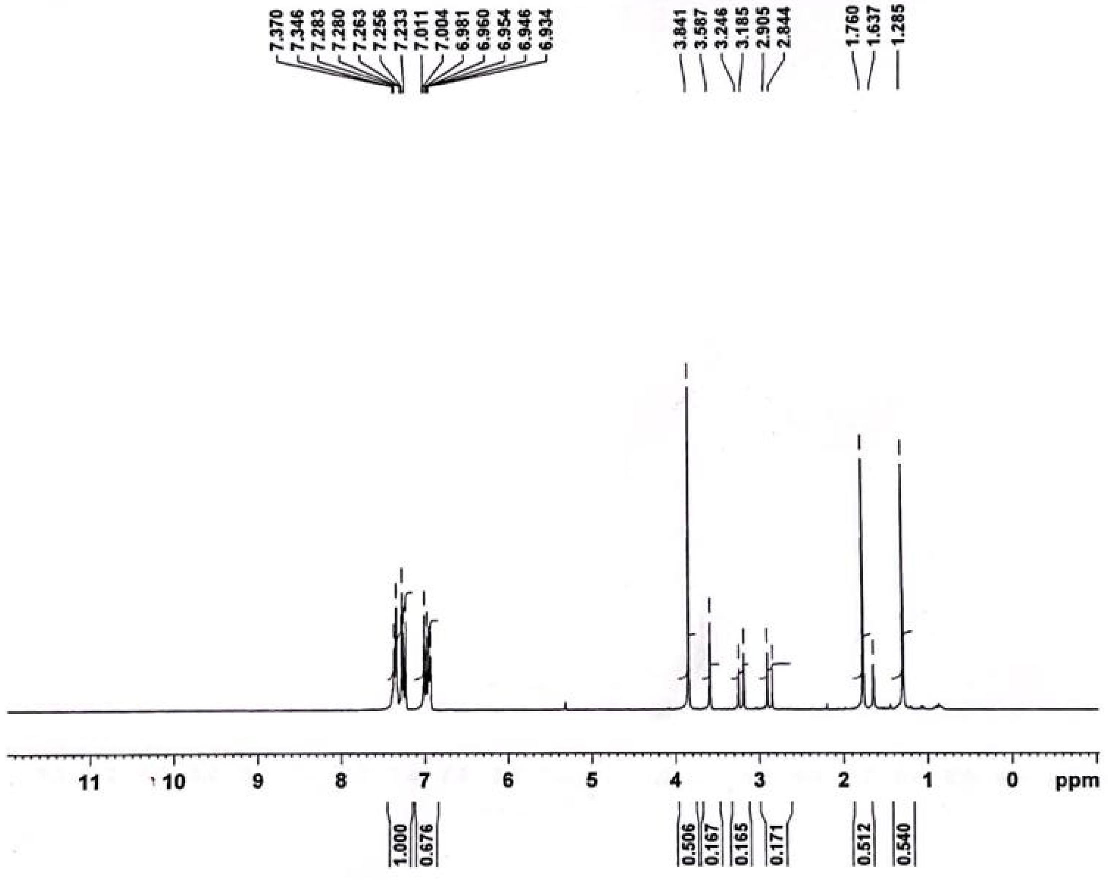

Figure S8: ^{13}C-NMR spectrum of compound **4c** recorded in $CDCl_3$.

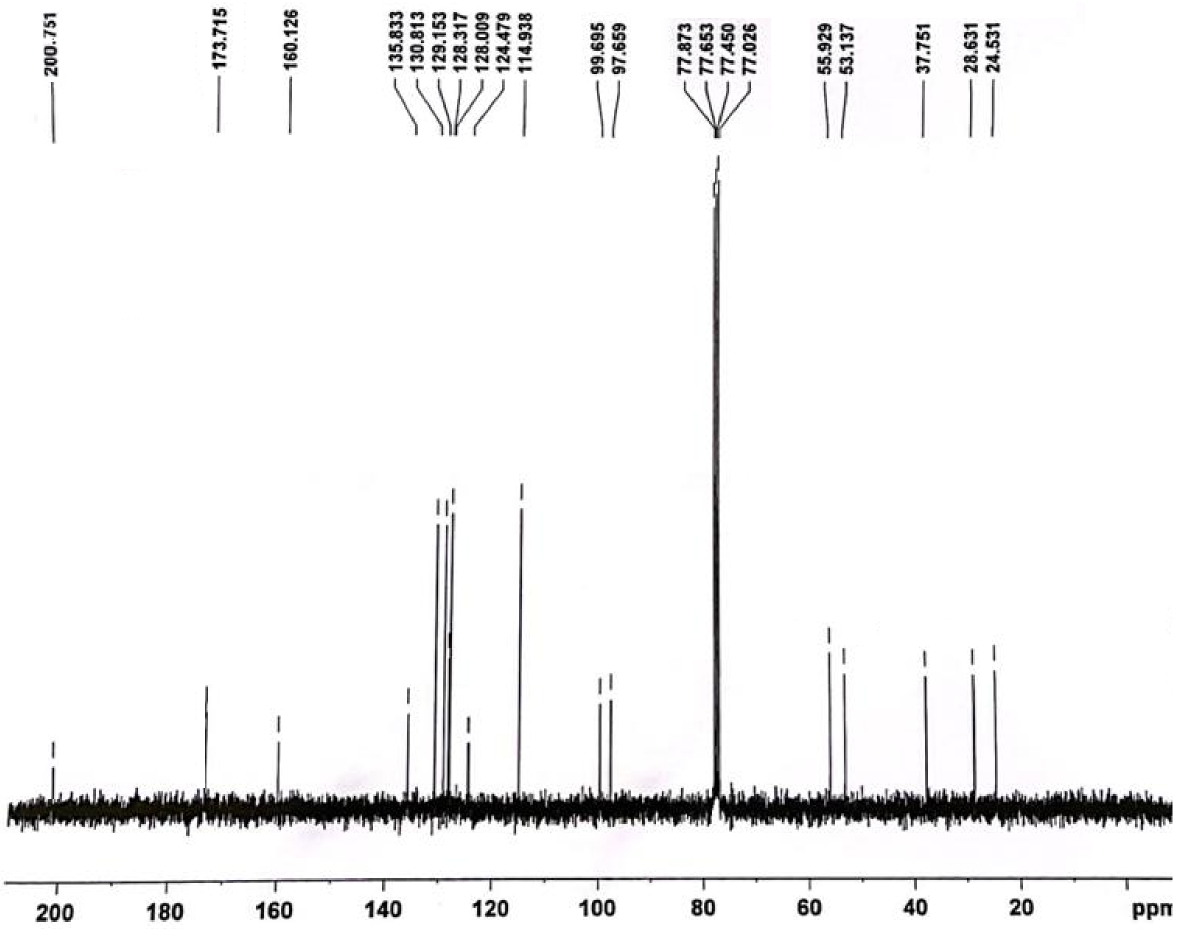

Figure S9: HRMS mass spectrum of compound **4c**.

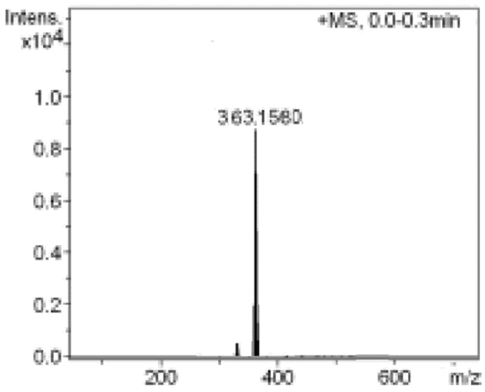

4,7-bis(4-methoxyphenyl)-3,3-dimethyl-1,2,7-triazaspiro[4.4]non-1-ene-8,9-dione (4d)

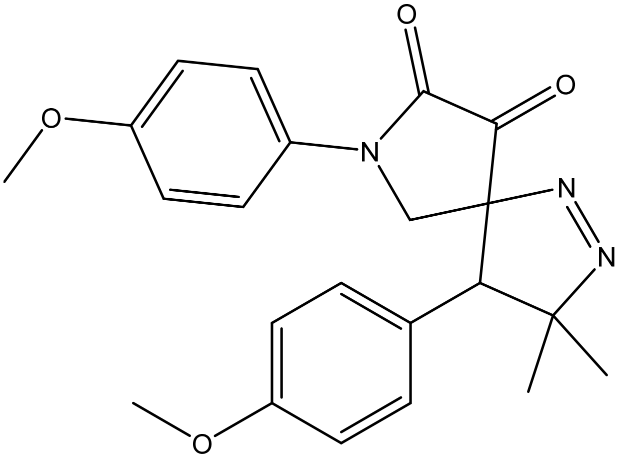

4d

Figure S10: ^{1}H-NMR spectrum of compound **4d** recorded in $CDCl_3$.

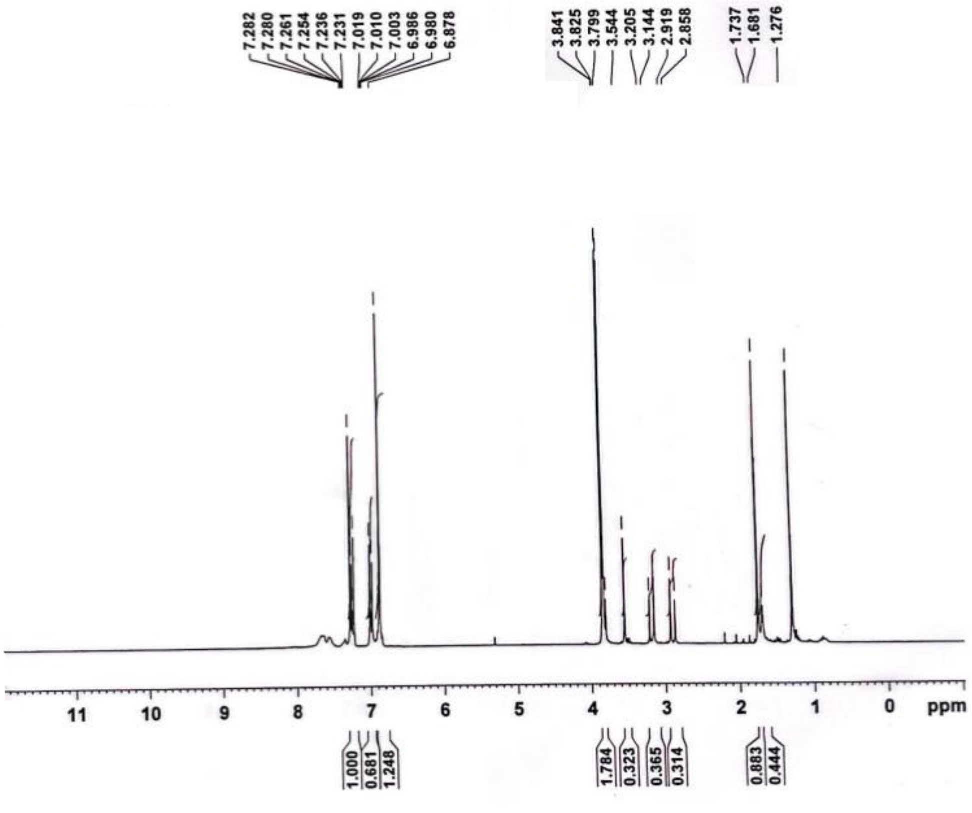

Figure S11: ^{13}C-NMR spectrum of compound **4d** recorded in CDCl3.

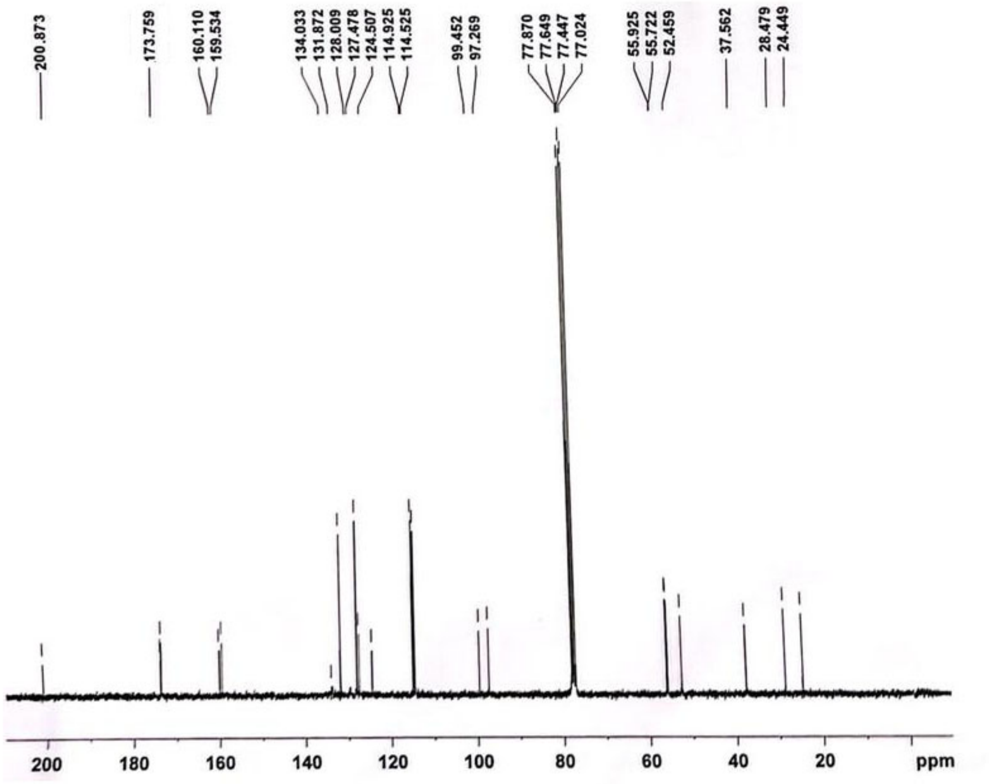

Figure S12: HRMS mass spectrum of compound **4d**.

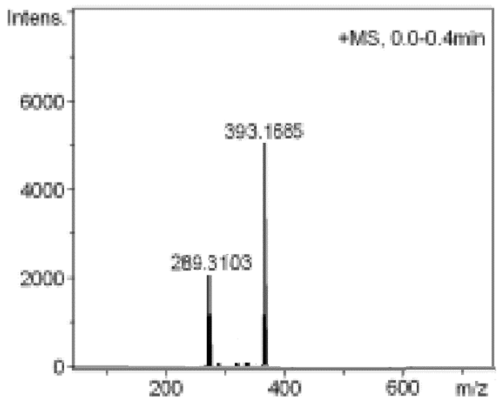

1,1-dimethyl-2,5-diphenyl-5-azaspiro[2.4]heptane-6,7-dione (5a)

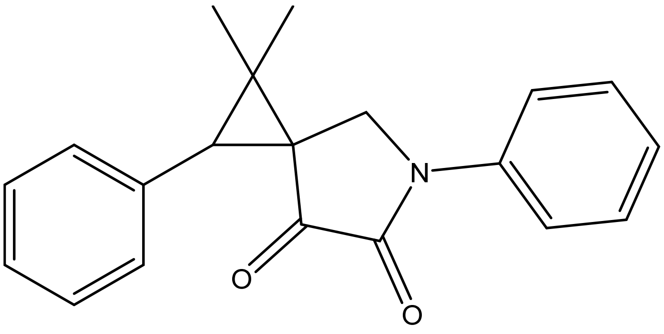

5a

Figure S13: ^{1}H-NMR spectrum of compound **5a** recorded in $CDCl_3$.

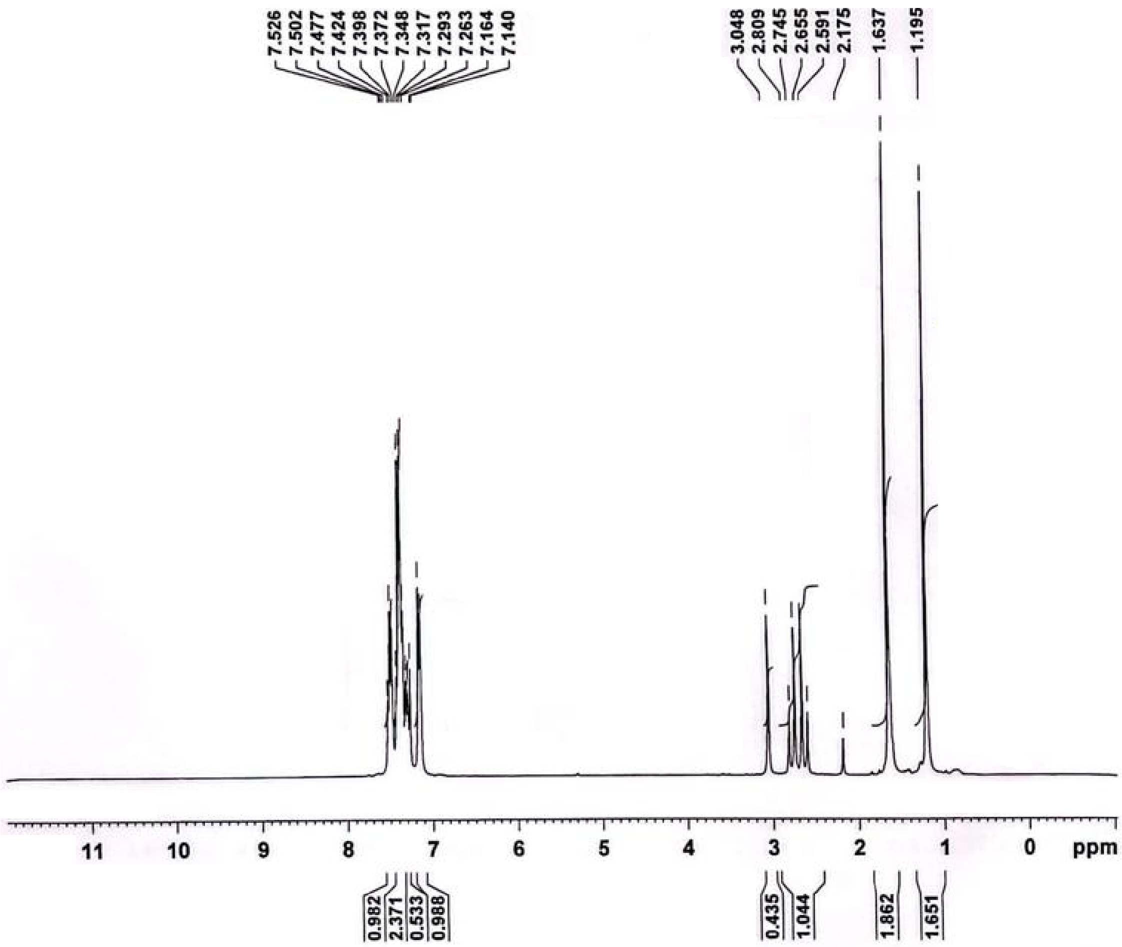

Figure S14: ^{13}C-NMR spectrum of compound **5a** recorded in $CDCl_3$.

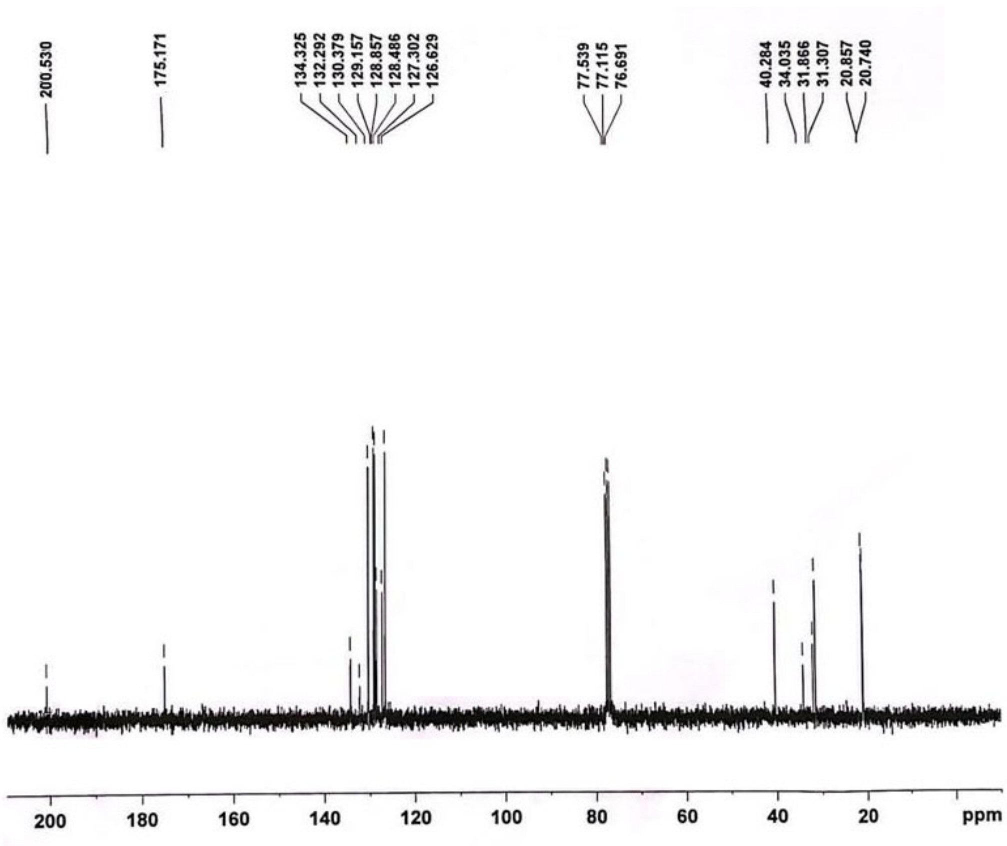

Figure S15: HRMS mass spectrum of compound **5a**.

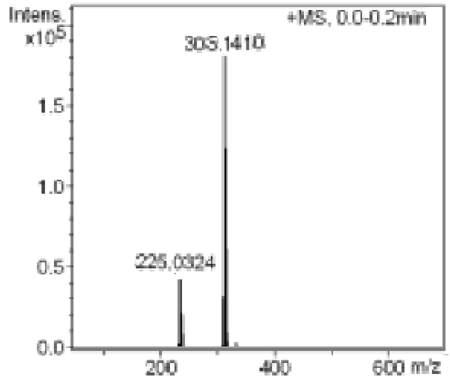

5-(4-methoxyphenyl)-1,1-dimethyl-2-phenyl-5-azaspiro[2.4]heptane-6,7-dione (5b)

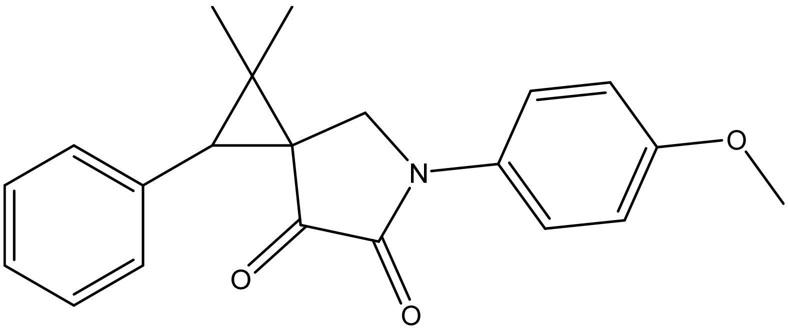

5b

Figure S16: ^{1}H-NMR spectrum of compound **5b** recorded in $CDCl_3$.

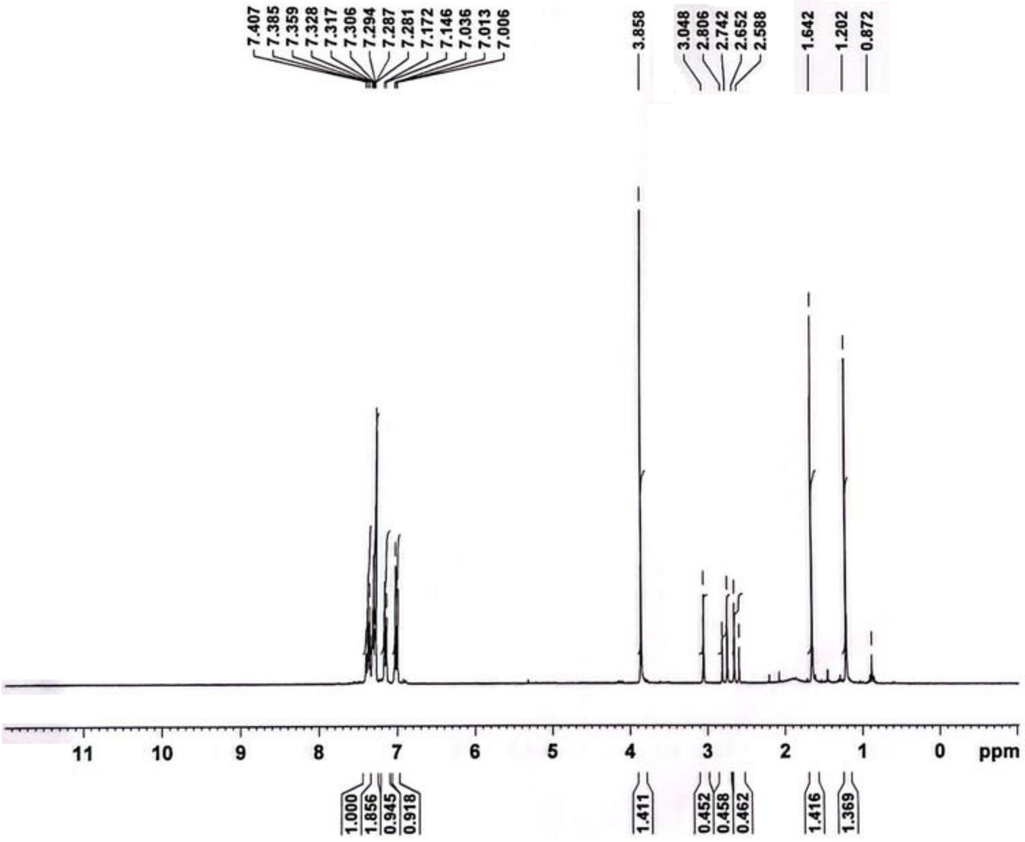

Figure S17: ^{13}C-NMR spectrum of compound **5b** recorded in $CDCl_3$.

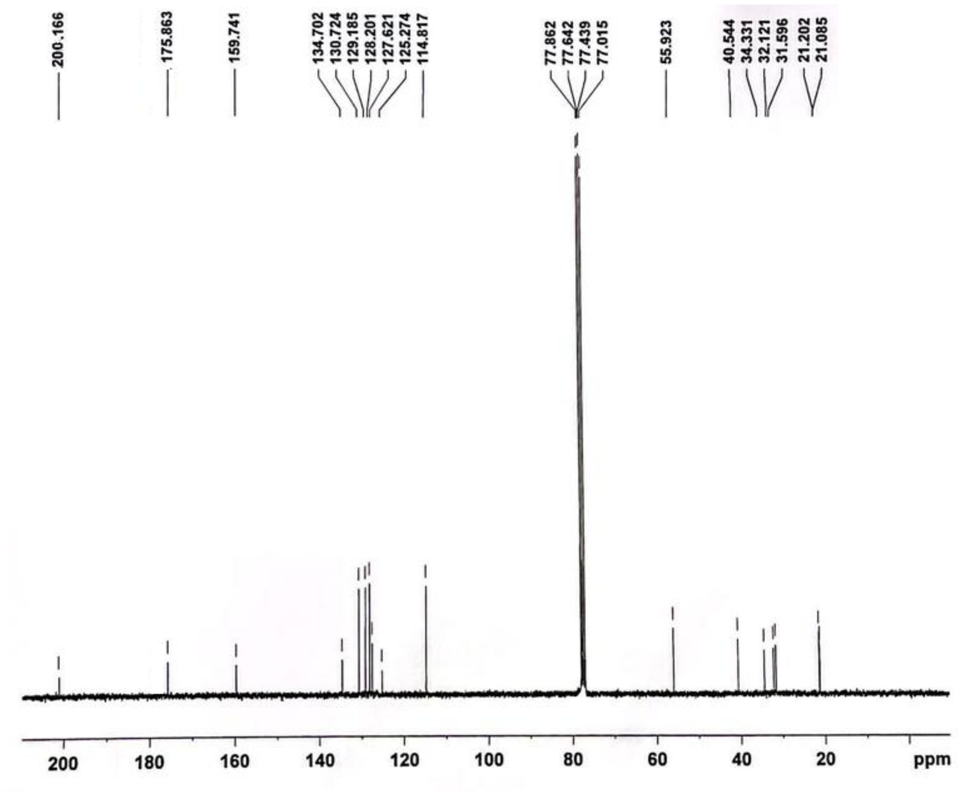

Figure S18: HRMS mass spectrum of compound **5b**.

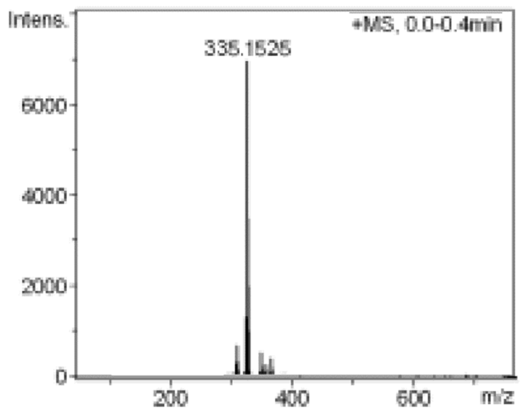

2-(4-methoxyphenyl)-1,1-dimethyl-5-phenyl-5-azaspiro[2.4]heptane-6,7-dione (5c)

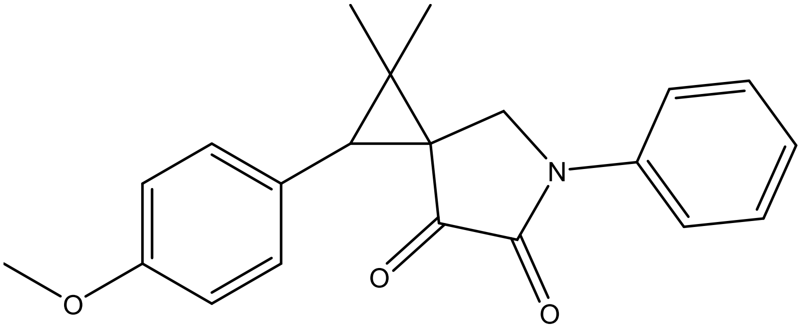

5c

Figure S19: ^{1}H-NMR spectrum of compound **5c** recorded in $CDCl_3$.

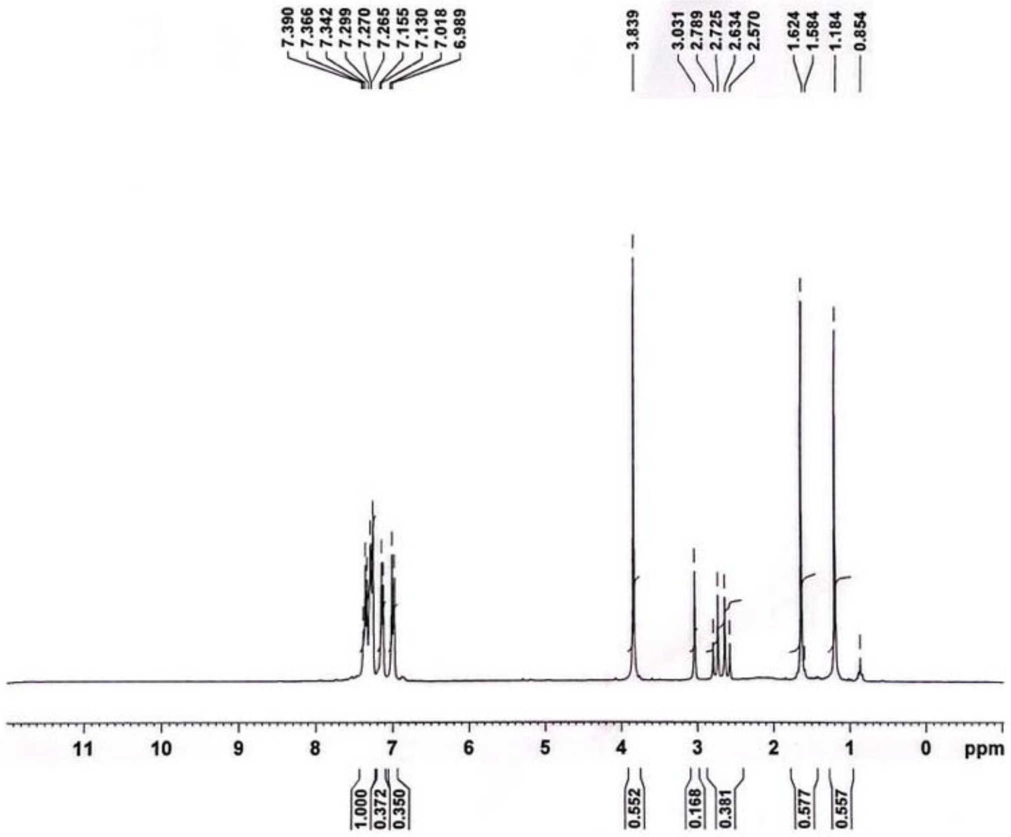

Figure S20: ^{13}C-NMR spectrum of compound **5c** recorded in $CDCl_3$.

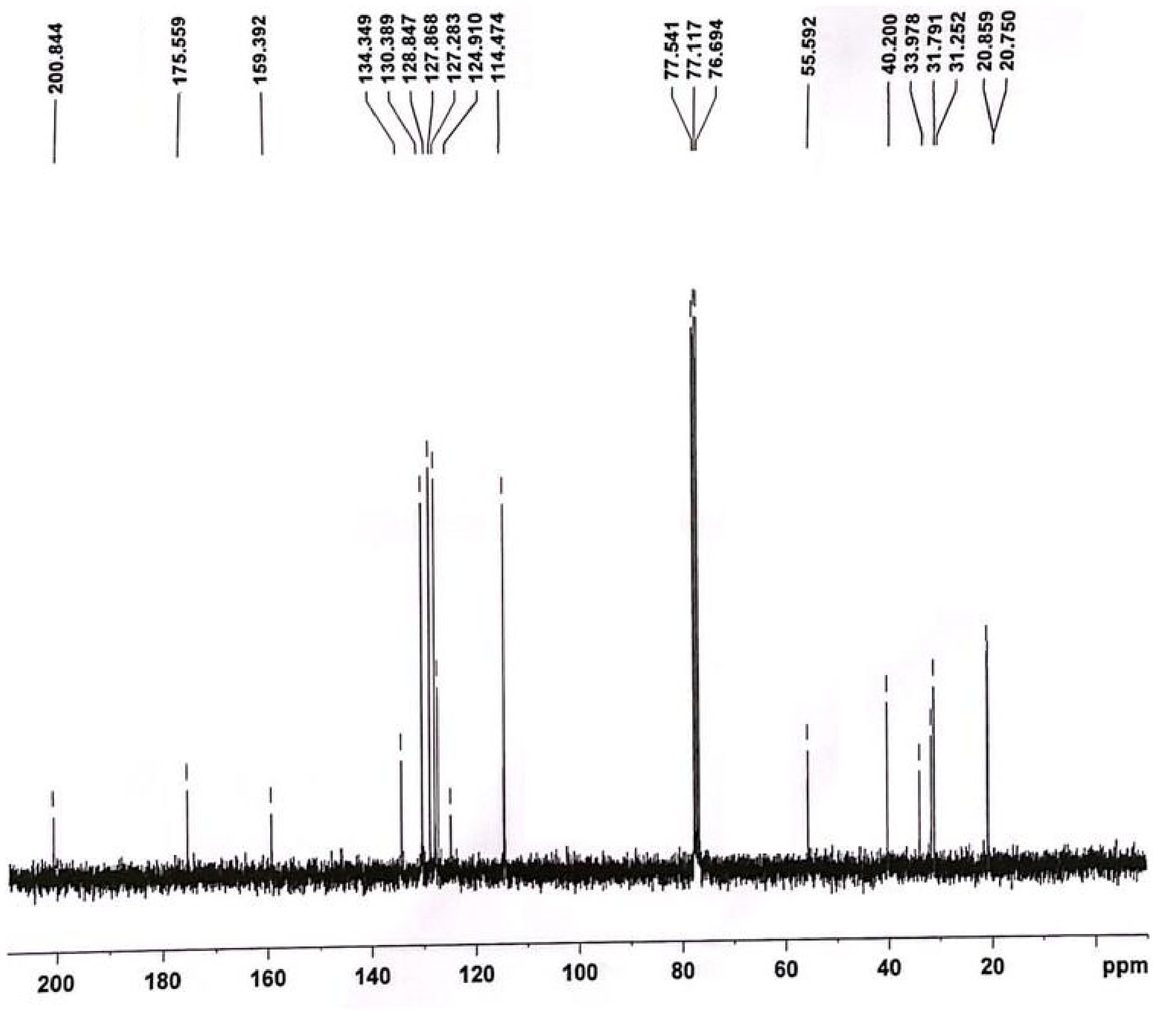

Figure S21: HRMS mass spectrum of compound **5c**.

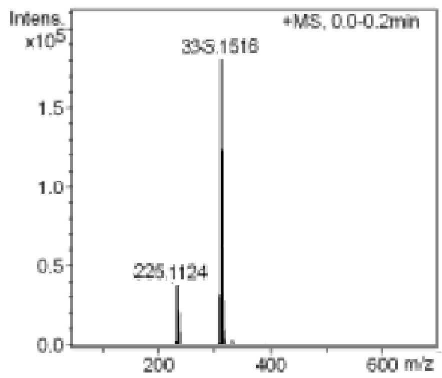

2,5-. bis(4-methoxyphenyl)-1,1-dimethyl-5-azaspiro[2.4]heptane-6,7-dione (5d)

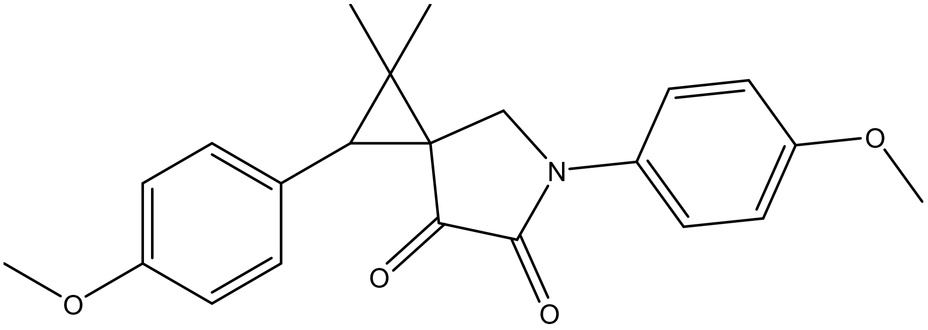

5d

Figure S22: ^{1}H-NMR spectrum of compound **5d** recorded in $CDCl_3$.

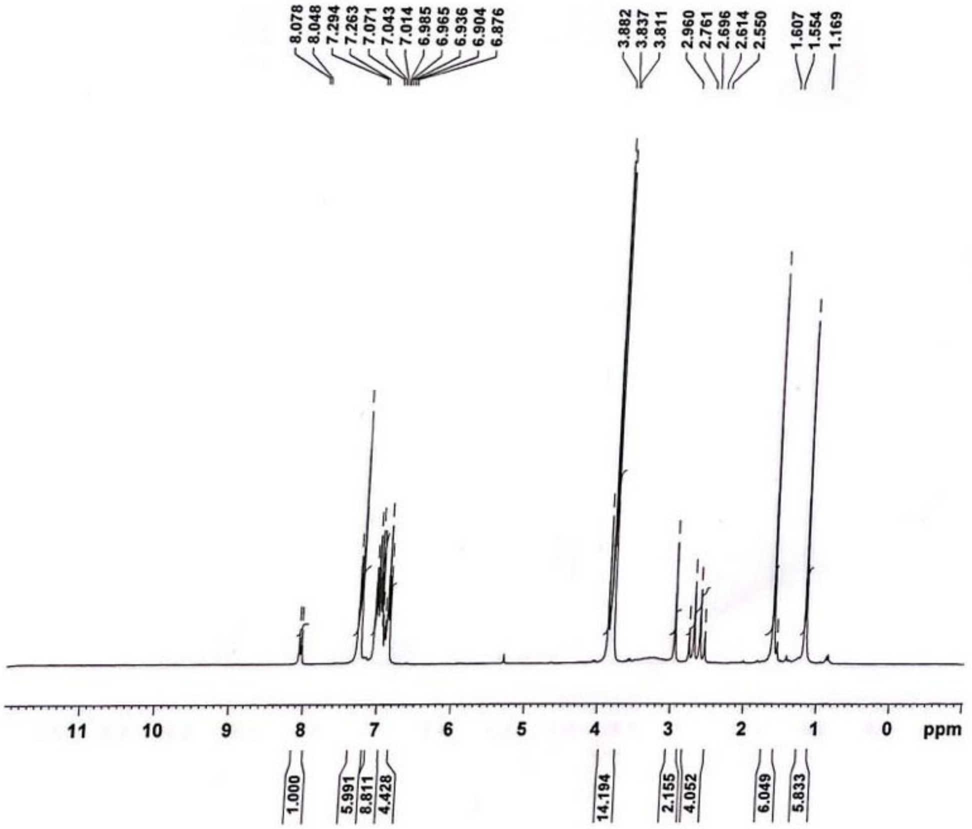

Figure S23: ^{13}C-NMR spectrum of compound **5d** recorded in $CDCl_3$.

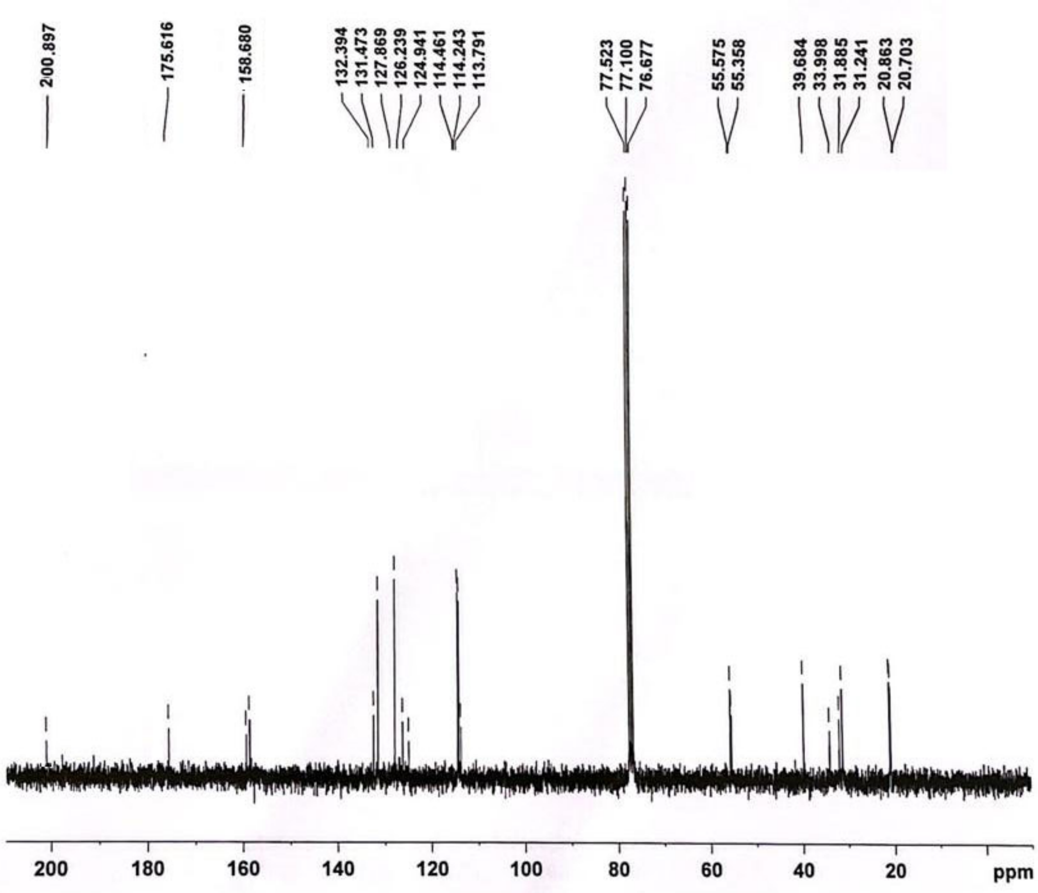

Figure S24: HRMS mass spectrum of compound **5d**.

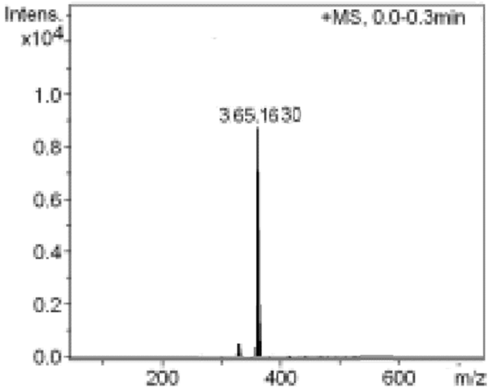

Supplement: Supplementary file 1 [file molecules-27-02470-s001.zip › molecules-1653131-Supplementary.pdf]
